# Mortality in people living with dementia who self-harmed: An Australian data linkage study

**DOI:** 10.1177/00048674241278243

**Published:** 2024-09-09

**Authors:** Adrian R Walker, Preeyaporn Srasuebkul, Julian N Trollor, Anne PF Wand, Brian Draper, Rachael C Cvejic, Annette Moxey, Simone Reppermund

**Affiliations:** 1 Department of Developmental Disability Neuropsychiatry, Faculty of Medicine & Health, UNSW Sydney, Sydney, NSW, Australia; 2 Centre for Big Data Research in Health, Faculty of Medicine & Health, UNSW Sydney, Sydney, NSW, Australia; 3 National Centre of Excellence in Intellectual Disability Health, Faculty of Medicine & Health, UNSW Sydney, Sydney, NSW, Australia; 4 Centre for Healthy Brain Ageing, Faculty of Medicine & Health, UNSW Sydney, Sydney, NSW, Australia; 5 Specialty of Psychiatry, Faculty of Medicine and Health, University of Sydney, Camperdown, NSW, Australia; 6 Discipline of Psychiatry & Mental Health, Faculty of Medicine & Health, UNSW Sydney, Sydney, NSW, Australia; 7 Older People’s Mental Health Service, Concord Centre for Mental Health, Sydney Local Health District, Concord, NSW, Australia; 8 Eastern Suburbs Older Persons Community Mental Health Service, Prince of Wales Hospital, Randwick, NSW, Australia; 9 Dementia Australia, Griffith, ACT, Australia

**Keywords:** Dementia, suicide, self-harm, epidemiology, mortality

## Abstract

**Objectives:**

This study aimed to examine mortality for people living with dementia/mild cognitive impairment who self-harmed.

**Methods:**

We conducted a retrospective cohort study in New South Wales, Australia, using data ranging from 2001 to 2015. From people who accessed hospital services in the study period, we identified 154,811 people living with dementia/mild cognitive impairment, 28,972 who self-harmed and 1511 who had a record of both dementia/mild cognitive impairment and self-harm. We examined rates, causes and predictors of death for people with dementia/mild cognitive impairment and/or self-harm diagnoses using flexible parametric survival analyses. We explored rates of repeat self-harm in people living with dementia who self-harmed.

**Results:**

Circulatory disorders accounted for 32.0% of deaths in people with a living with dementia who self-harmed, followed by neoplasms (14.7%), and mental and behavioural disorders (9.6%). Death was more likely for someone who had self-harmed if they developed dementia/mild cognitive impairment. Predictors of death included male sex, greater physical comorbidity, a history of delirium, more previous emergency department presentations and fewer previous mental health ambulatory service days. Greater engagement with outpatient mental health services predicted a decreased likelihood of repeat self-harm.

**Discussion:**

We found that mortality increases when people who self-harm develop dementia. We argue post-diagnosis support offers a potential opportunity to reduce mortality rates in people with both dementia and self-harm diagnoses.

## Introduction

The burden of poor mental health and suicide in older people is substantial (Draper, 2015; Murphy et al., 2012). In the 2017/2018 financial year in Australia, 17.3% of men and 22.4% of women over 65 reported having a current long-term mental or behavioural health disorder (Australian Institute of Health and Welfare (AIHW), 2020). These numbers are elevated for people living with dementia, with a large study from the US finding that around 25% of people living with dementia (98% male in their study) had at least one mental health disorder in the 2012/2013 financial year (Lai et al., 2018). Furthermore, poor mental health is a risk factor for self-harm in people living with dementia (Walker et al., 2023), with increased rates of self-harm in people living with dementia compared to the general population after adjusting for age (Mitchell et al., 2017). This increased rate of self-harm is concerning, particularly given that older adults living with dementia who self-harmed are 67 times more likely to die from suicide than the general population of older adults (Murphy et al., 2012).

More broadly, people living with dementia experience increased rates of all-cause mortality compared to the general population, with a 2013 review finding death is 1.4–5.2 times more likely to occur at any given point in time compared to people without dementia (Todd et al., 2013). The most prevalent underlying causes of death for people living with dementia are circulatory and respiratory disorders (Brunnström and Englund, 2009; Garcia-Ptacek et al., 2016), with dementia also often reported as the underlying cause of death (Garcia-Ptacek et al., 2016).

Given the high rate of self-harm in people living with dementia, and the fact that self-harm is associated with a substantial increase in the likelihood of death (particularly in older adults; Bergen et al., 2012; Hawton et al., 2006), understanding when and why death occurs for people living with dementia who self-harmed may inform the broader care approach for people living with dementia generally. However, limited research has investigated the patterns and risk factors of mortality in this population.

The current study aimed to address this research gap by exploring patterns and predictors of mortality for people living with dementia who have self-harmed. Specifically, we were interested in investigating the rates and causes of mortality for people living with dementia who had self-harmed compared to people living with dementia (who had not self-harmed) and people who had self-harmed (but were not living with dementia). We were also interested in finding potential predictors of mortality for people living with dementia who self-harmed.

To meet these aims, we leveraged two previously defined cohorts of people (one of people who presented to hospital and were coded as having a dementia diagnosis, and one of people who presented to hospital and received a diagnosis of self-harm) from New South Wales (NSW), Australia, between 2001 and 2015 (Walker et al., 2023). We defined self-harm broadly as all codes indicating intentional self-harm captured in hospital data, irrespective of if these were in the context of a suicide attempt. To assess predictors of mortality, we developed a directed acyclic graph (DAG) (Greenland et al., 1999) that outlines the theorised causal relationships between variables in our study. Choice of variables and relationships represented in the DAG was determined by the variables available in the data linkage, previous research and discussions within the research team. This DAG was used to identify confounder variables for adjustment when modelling predictors of mortality.

## Methods

### Dementia advocate group

We consulted with 10 lived experience dementia advocates (including both people living with dementia and carers) throughout the project. In a series of meetings, advocates provided feedback on our analysis plan (including predictor variables to include in any modelling) and interpretation of our results. All advocates were given the opportunity to read and comment on the manuscript prior to submission for publication.

### Data sets and record linkage

We derived the cohort for this study from an existing linkage of individuals from NSW Australia, who had either received government services or hospital care for an intellectual disability, or had received hospital care for a neuropsychiatric disorder (including both dementia and self-harm) (Reppermund et al., 2019).

We used the following data sets from this linked cohort for our study:

- The Admitted Patient Data Collection (APDC) containing information on all hospital admission in NSW between 1 July 2001 and 31 December 2015;
- The Emergency Department Data Collection (EDDC) containing information on most emergency department presentations in NSW between 1 January 2005 and 31 December 2015;
- The Mental Health Ambulatory Data Collection (MH-AMB) containing information on all activities in NSW public ambulatory mental health services between 1 January 2001 and 31 December 2015;
- The NSW Registry of Births, Deaths and Marriages (RBDM) containing information on all dates of death in NSW between 1 January 1994 and 31 December 2015.
- The NSW Cause of Death Unit Record File (COD-URF) containing information on all causes of death in NSW between 1 January 1985 and 31 December 2013.

A comprehensive description of each data set is provided in the work of Reppermund et al. (2019).

Deidentified linkage of the data sets was performed by the NSW Centre for Health Record Linkage based on a statistical linkage key (SLK581) and unit record data were sent to researchers after removal of personal identifiers.

### Study population

The cohorts for this study were drawn from the cohorts described in the work of Walker et al. (2023). Briefly, we formed two cohorts of people aged over 40:

- People without a history of self-harm who presented to hospital with a diagnosis of dementia or mild cognitive impairment between 1 July 2001 and 31 December 2014 (the dementia cohort, *n* = 154,811).
- People without a history of dementia who presented to hospital for self-harm between 1 January 2005 and 31 December 2014 (the self-harm cohort, *n* = 28,972).

The presence of dementia and self-harm was defined using the criteria described in the work of Walker et al. (2023). Briefly, dementia and mild cognitive impairment were identified from International Classification of Diseases, tenth revision (ICD-10) codes from inpatient hospital admissions (APDC) and outpatient mental health contacts (MH-AMB). These codes included A81.0, F00-F03, F06.7, G30, G31 and F05.1. Self-harm was identified through the presence of relevant ICD-10 (X60-X84 and Y87.0), ICD-9 (E950-E959) or relevant Systematized Nomenclature of Medicine (SNOMED; codes identified in the work of Chitty et al. (2022)) codes recorded during inpatient hospital admissions (APDC), outpatient mental health contacts (MH-AMB) and emergency department (EDDC) presentations. These codes capture a variety of reasons for self-harm: ICD-10 includes all forms of intentional self-harm; ICD-9 codes include cases of self-inflicted injury (irrespective of intent); SNOMED codes were captured as those that clearly indicated self-inflicted injury/poisoning or suicidal behaviour (as per Chitty et al. (2022)). Patients who presented with both self-harm and dementia for the first time on the same day were placed in the dementia cohort (as it is likely dementia symptoms were present prior to this first presentation).

From these two cohorts, we defined a subgroup of people who had a subsequent diagnosis of self-harm in the dementia cohort, or a subsequent diagnosis of dementia in the self-harm cohort between 1 January 2005 and 31 December 2015 (the dementia and self-harm subgroup). People entered this subgroup on:

- The date of their first diagnosis of self-harm if they were in the dementia cohort (*n* = 569).
- The date of their first diagnosis of dementia if they were in the self-harm cohort (*n* = 839).
- The first date of entry to the dementia cohort if they also had a recorded diagnosis of self-harm (*n* = 206).

To ensure that we had adequate follow-up time for analysis in this subgroup, the subgroup excluded people who died on the day they received relevant diagnoses for inclusion to the subgroup (*n* = 73; 42 from dementia cohort).

### Predictor variables

We used all the predictor variables previously defined in the work of Walker et al. (2023). These included the following:

- Sex;
- Aboriginal or Torres Strait Islander status (our ethics approval only covered using the status as a control variable, hence, results omitted);
- Index of relative socioeconomic disadvantage (IRSD), which indicates the relative socioeconomic disadvantage of an individual’s location of residence, split into quintiles (Australian Bureau of Statistics (ABS), 2018b);
- Remoteness of residential address, which indicates the rurality of an individual’s location of residence (Australian Bureau of Statistics (ABS), 2018a).

These variables were determined by Reppermund et al. (2019) by combining the available data sets and determining the most likely value for each variable and were considered fixed for the study period. We also included eight additional time-varying variables based on the data recorded in the APDC, EDDC and MH-AMB. These included the following:

- Age;
- Marital status;
- Number of involuntary mental health admissions to hospital in the previous year;
- Number of emergency department presentations in the previous year;
- Number of Elixhauser comorbidities recorded in the previous year, excluding codes associated with mental health (as they were included as separate predictors). Elixhauser et al.’s (1998) comorbidities are a list of conditions associated with increased likelihood of mortality;
- Number of mental health ambulatory days (i.e. days in contact with a community mental health team) in the previous year;
- A diagnosis in hospital of certain mental health conditions, including depression, drug and alcohol abuse, psychotic disorders, anxiety, organic disorders, delirium, behavioural problems and personality disorders, with each the presence/absence of each disorder entered as a separate predictor into the model;
- Whether someone had entered the dementia and self-harm subgroup.

Unlike the fixed variables, the value for these variables changed over time depending on the observed data. The specific method for determining these time-varying variables (and the calculation of yearly periods) is detailed in the work of Walker et al. (2023).

### Outcome variables

The main outcome in this study was death, determined by a recorded date of death in the RBDM. One analysis also included hospital contacts (including inpatient admissions, emergency department presentations or outpatient mental health contacts) for self-harm as an outcome, determined using the codes outlined for entry to the self-harm cohort outlined in the ‘Study population’ section.

### Statistical analysis

For both the dementia cohort and self-harm cohorts, we calculated the rate of death per 1000 person years in the study. We calculated the rate of death for the dementia and self-harm subgroup separately, removing any contributing person-time after entering the dementia and self-harm subgroup. We also calculated the frequency of different underlying causes of death by ICD-10 chapter (splitting out death due to suicide [ICD10 codes X60-X84] into a separate category) for the dementia cohort and self-harm cohort up until 31 December 2013 (the end of the capture period for our cause of death data), calculating the frequency for those that entered the dementia and self-harm subgroup separately.

We then examined the rates of death for those who entered the dementia and self-harm subgroup compared to those who did not, using flexible parametric survival analyses (Royston and Lambert, 2011). One set of analyses examined rates of death for people in the dementia cohort, comparing those with and without a diagnosis of self-harm. The second set of analyses compared rates of death for people in the self-harm cohort, comparing those with and without a diagnosis of dementia. For both cohorts, we conducted k-nearest propensity score matching without replacement using the MatchIt package in R (Stuart et al., 2011). We matched those who entered the dementia and self-harm subgroup with those who had not on age at first dementia presentation (for the dementia cohort) or self-harm presentation (for the self-harm cohort). We fit four models for each cohort using one-nearest matching, five-nearest matching, 20-nearest matching and the entire cohort. People were entered into the models at their date of entry into either the dementia cohort or self-harm cohort or 1 January 2005 (whichever occurred last). For all analyses in this paper, people were censored on either 31 December 2015, or at the date of their outcome (death), whichever occurred first. Age (bounded between 40 and 120 years) was used as the timeline variable.

Following these analyses, we examined predictors of death for those who entered the dementia and self-harm subgroup. We first plotted the time to death following entry to the subgroup. We then used flexible parametric survival analyses with three splines to model predictors of death for people in the dementia and self-harm subgroup. In models analysing the dementia and self-harm subgroup specifically, people were entered at their date of entry into either the dementia and self-harm subgroup or 1 January 2005 (whichever occurred last), and as above people were censored on either 31 December 2015, or at the date of their outcome (death).

**Figure 1.**
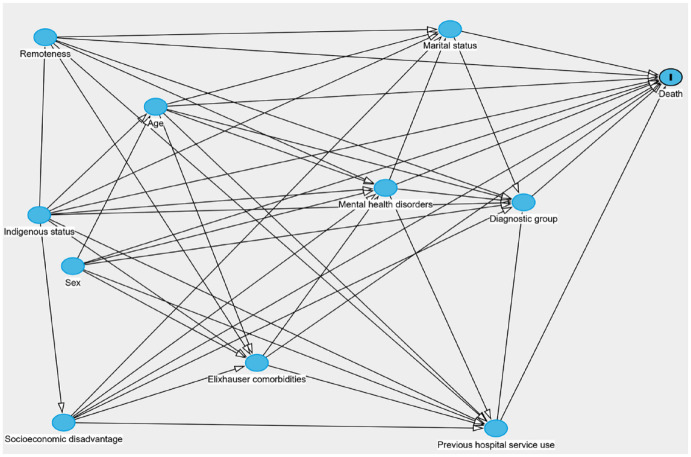
Directed acyclic graph showing proposed causal relationships between our predictor variables and the death. Figure made using DAGitty (www.dagitty.net/) (Textor et al., 2017).

As in the work of Walker et al. (2023), we ran both a fully adjusted model and several individually adjusted models. Our individually adjusted models were adjusted according to our proposed causal links given in the DAG in Figure 1 (Greenland et al., 1999; Walker et al., 2023). Briefly, the approach involves first identifying plausible causal pathways (shown in the DAG) between the different variables available in our linkage and then adjusting only for confounding variables (those that are thought to causally influence both the exposure and outcome). This approach allows us to estimate the *total effect* of the relevant predictor variable on mortality, that is, the effect of the predictor on the outcome including the effect of any mediating variables.

Finally, as a history of self-harm may indicate an increased risk of death from suicide, and as our previous work has shown that for people living with dementia self-harm is strongly predicted by poor mental health (Walker et al., 2023), we were interested in assessing whether engagement with outpatient mental health services decreased the risk of future self-harm following entry into the dementia and self-harm subgroup. To conduct this assessment, we ran a flexible parametric survival analysis with three splines with competing risks where death was a competing event. The main outcome of interest was contact with the hospital for self-harm or suicide. People could have multiple occurrences of the self-harm outcome in our analysis. As we were only interested in the effect of previous mental health ambulatory use on future self-harm, we adjusted for confounders based on the DAG in Figure 1. For comparison, we also fit a model including all variables.

Analysis was conducted using a combination of R 4.1.2, Stata 17 and SAS 9.4 (R Core Team, 2021; SAS Institute, Inc., 2016; StataCorp, 2021). For all analyses, we ran a sensitivity analysis excluding mild cognitive disorder (ICD10 code F06.7) as an indicator for dementia (Supplemental material 1).

### Ethics approval

The study was approved by the NSW Population and Health Services Research Ethics Committee (HREC/13/CIPHS/7; Cancer Institute NSW reference: 2013/02/446 and Sub-study Reference number: 2019/ETH01806). This approval included a waiver of informed consent.

## Results

### Comparison of mortality between cohorts

Those in the dementia cohort had a rate of mortality of 234.0 per 1000 person years, while those in the self-harm cohort had a rate of mortality of 20.2 per 1000 person years (excluding person-time for those who entered the dementia and self-harm subgroup). Those in the dementia and self-harm subgroup had a rate of mortality of 129.2 per 1000 person-years.

Table 1 shows the recorded causes of death for people in the dementia cohort, self-harm cohort and dementia and self-harm subgroup separately.

**Table 1.** Causes of death by International Classification of Diseases, tenth revision (ICD-10) for people in the dementia and self-harm subgroup, ordered by most to least common.

International Classification of Diseases, tenth revision (ICD-10) Chapter	Frequency (%)
Total	456
Circulatory	146 (32.0)
Neoplasms	67 (14.7)
Mental and behavioural	44 (9.6)
Respiratory	43 (9.4)
Nervous	39 (8.6)
Digestive	32 (7.0)
External causes (not self-harm)	25 (5.5)
Endocrine	20 (4.4)
Certain infectious and parasitic diseases	16 (3.5)
External causes (self-harm)	10 (2.2)
Genitourinary	9 (2.0)
Musculoskeletal	$
Signs and symptoms	$

ICD: International Classification of Diseases.

$: Data censored due to small cell size

**Figure 2.**
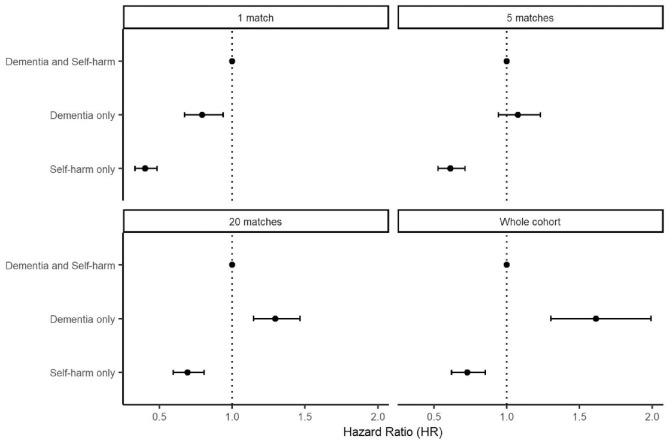
Hazard ratios for death, with those in the dementia and self-harm subgroup as the baseline. Error bars represent 95% confidence intervals.

Adjusted hazard ratios from our flexible parametric survival analysis can be seen in Figure 2 (full regression results can be seen in Supplemental material 2). People in the dementia cohort (excluding those who self-harmed) had the highest rate of death (113,590 deaths overall, 73.4% of cohort), those in the self-harm cohort had the lowest rate (2905 deaths overall, 10.0% of cohort) and those in the dementia and self-harm subgroup fell in between (681 deaths overall, 42.2% of subgroup). This pattern of results was maintained in our survival analyses of the self-harm cohort and when using 20-nearest matching, but disappeared when using five-nearest matching and reversed when using one-nearest matching for the dementia cohort.

### Mortality for those in the dementia and self-harm subgroup

**Figure 3.**
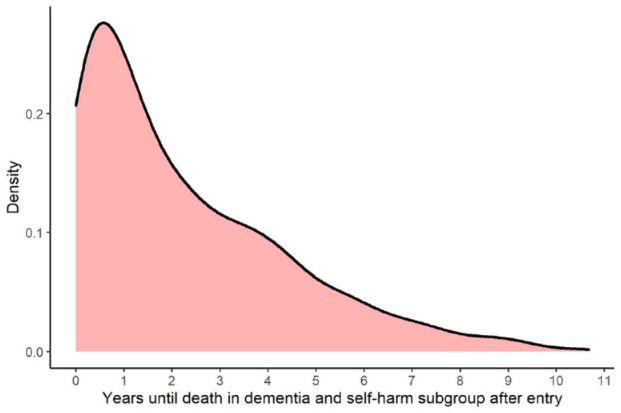
Density of time to death after entry into the dementia and self-harm subgroup.

Overall, 681 people (42.2%) in the dementia and self-harm subgroup died in the study period, with 456 dying before 1 January 2014 and having a recorded cause of death. Figure 3 shows the density of time to death for people who died in the study period following entry into the dementia and self-harm subgroup. Around half (55.7%) of all deaths occurred within the first 2 years after entry into the subgroup. Overall, around 23% of people with dementia died within 2 years of self-harming (if they were previously living with dementia) or being diagnosed with dementia (if they had previously self-harmed), with 19% of people dying after 2 years.

Table 2 shows the output of the flexible parametric survival analyses examining time to death in the dementia and self-harm subgroup. In both sets of models, men had a higher likelihood of death than women. Furthermore, a higher number of Elixhauser comorbidities and emergency department presentations in the previous year were associated with an increased likelihood of death. Mental health ambulatory use in the previous year was associated with a decreased likelihood of death. A history of delirium was associated with an increased likelihood of death. A history of drug and alcohol abuse was associated with an increase in the likelihood of death in the individually adjusted model, but not in the model with all variables included. Remoteness was associated with an increased rate of death in the model with all variables included, but not in the individual adjusted model. Marital status was a significant predictor in both sets of models, although this appeared to be driven by those in the ‘Unknown’ marital status category (numerical output excluded due to lack of interpretability).

**Table 2.** Predictors of death for people in the dementia and self-harm subgroup with age as the time scale.

Variable	Variables individually adjusted according to causal model			All variables included model		
	Incident rate ratio (95% CI)	Standard error	*p*	Incident rate ratio (95% CI)	Standard error	*p*
Sex (female)^†^	0.69 (0.59, 0.81)	0.05	<0.001	0.74 (0.63, 0.87)	0.06	<0.001
Remoteness^†^			0.564^$^			0.034^$^
Major cities (reference)	1	–	–	1	–	–
Inner regional	0.96 (0.79, 1.16)	0.09	0.639	1.02 (0.83, 1.26)	0.11	0.860
Outer regional and beyond	1.17 (0.83, 1.66)	0.21	0.367	1.58 (1.12, 2.24)	0.28	0.009
Index of relative socioeconomic disadvantage quintile^%^			0.544^$^			0.611^$^
1–2 (Most disadvantaged)	1	–	–	1	–	–
3–4	0.92 (0.74, 1.13)	0.10	0.42	0.91 (0.74, 1.12)	0.10	0.378
5–6	0.85 (0.67, 1.07)	0.10	0.157	0.86 (0.68, 1.09)	0.11	0.214
7–8	0.97 (0.75, 1.24)	0.12	0.778	0.99 (0.76, 1.28)	0.13	0.923
9–10 (Least disadvantaged)	1.03 (0.82, 1.29)	0.12	0.817	1.04 (0.82, 1.32)	0.13	0.737
Marital status^%^			<0.001^$^			<0.001^$^
Married/de facto	1	–	–	1	–	–
Divorced/widowed/separated	0.93 (0.80, 1.09)	0.07	0.373	0.92 (0.79, 1.08)	0.07	0.328
Never married	1.08 (0.80, 1.45)	0.16	0.622	1.07 (0.79, 1.44)	0.16	0.652
Num. Elixhauser comorbidities in year prior	1.22 (1.17, 1.26)	0.02	<0.001	1.17 (1.13, 1.22)	0.02	<0.001
Mental health ambulatory use in year prior (per 10 days)	0.91 (0.83, 0.99)	0.04	0.028	0.89 (0.81, 0.98)	0.04	0.016
Involuntary mental health admissions in year prior (per 10 admissions)	1.01 (0.98, 1.03)	0.01	0.649	1.01 (0.99, 1.03)	0.01	0.452
Emergency department presentations in year prior (per 10 presentations)^§^	1.17 (1.03, 1.32)	0.07	0.013	1.20 (1.05, 1.35)	0.08	0.005
History of depression	1.00 (0.86, 1.17)	0.08	0.968	1.02 (0.86, 1.21)	0.09	0.844
History of drug or alcohol abuse	1.22 (1.03, 1.45)	0.11	0.025	1.20 (0.99, 1.45)	0.12	0.058
History of psychotic disorder	1.10 (0.93, 1.30)	0.09	0.250	1.07 (0.89, 1.28)	0.10	0.481
History of anxiety disorder	0.97 (0.83, 1.13)	0.08	0.682	0.94 (0.80, 1.11)	0.08	0.468
History of delirium	1.57 (1.32, 1.86)	0.14	<0.001	1.56 (1.31, 1.85)	0.14	<0.001
History of behavioural problems	1.14 (0.97, 1.33)	0.09	0.115	1.09 (0.91, 1.31)	0.10	0.338
History of personality disorders	0.85 (0.68, 1.07)	0.10	0.168	0.77 (0.60, 0.99)	0.10	0.041

CI: confidence interval.

$ Wald’s test for variable inclusion.

% ‘Unknown’ category excluded from output.

§ To allow for model convergence, if someone had over 100 presentations to ED in a single year, we treated them as having at most 101 presentations in that year.

### Repeat self-harm in the dementia and self-harm subgroup

Overall, 174 people either returned to hospital with a diagnosis of self-harm or had a cause of death recorded as self-harm in our study period. In our individually adjusted model, for every 10 mental health ambulatory days in the previous year, the incident rate ratio for self-harm in the dementia and self-harm subgroup decreased by 7% (95% confidence interval = 2%, 12%). That is, every 10 mental health ambulatory days in the previous year decreased the likelihood of self-harm by about 7%. Similarly, our model with all variables showed that for every 10 mental health ambulatory days in the previous year, the incident rate ratio for self-harm in the dementia and self-harm subgroup decreased by 8% (95% confidence interval: 3%, 13%). Supplemental material 3 contains the full regression results.

## Discussion

### Summary of findings

The current study explored patterns and predictors of mortality for people with dementia and/or self-harm diagnoses. We found people diagnosed with dementia with no record of self-harm had the highest rate of death in our study period, followed by people diagnosed with both dementia and self-harm and then people diagnosed with self-harm without dementia. This difference in mortality was maintained for the self-harm cohort when adjusting for potential confounders but reversed under conservative matching criteria for the dementia cohort.

Descriptive analysis suggested that people living with dementia who self-harmed had a mortality profile between (both for rates and causes) that of the dementia cohort and self-harm cohort. This difference likely reflects the relative age of the different groups. Those living with dementia who self-harmed had a median age of around 72 at entry to the dementia and self-harm subgroup. By contrast, the median age of entry was around 46 in the self-harm cohort and 84 in the dementia cohort (Walker et al., 2023). Given the age disparity between the different cohorts, it is not surprising that rates and causes of mortality differed.

In general, psychiatric profile did not predict mortality in the dementia and self-harm cohort. Previous research has suggested that attempted suicide in people living with dementia is not moderated by psychiatric comorbidities (Günak et al., 2021). The current study suggests that this result may apply more broadly to all-cause mortality, with some caveats. Our modelling showed those with a history of delirium died at a greater rate, consistent with previous literature on dementia and delirium (Witlox et al., 2010), and there was some evidence that a history of drug and alcohol abuse was associated with a greater rate of death, consistent with evidence on causes of mortality in people with mental health disorders (Plana-Ripoll et al., 2019). Those with more mental health ambulatory days in the previous year had a lower rate of death. Repeat self-harm in the dementia and self-harm subgroup was around 12% of the cohort, in line with previous studies on repeat self-harm identified in hospital data (Carroll et al., 2014), with prior mental health ambulatory use predicting a decrease in repeat self-harm (noting this decrease was not observed when we do not consider mild cognitive disorder as an indicator of dementia).

Overall, the estimated strength of the predictors of death in the dementia and self-harm subgroup was modest, with most showing an incidence rate ratio between 0.8 and 1.2. Positively, our models demonstrate good precision to uncover these smaller effects. Furthermore given the substantially increased risk of death for people living with dementia compared to the age-matched general population (Guehne et al., 2005), even a small change in the risk of death for populations living with dementia can be clinically relevant. As such, although we caution against a strong interpretation of these modest effects, we argue the findings remain clinically meaningful.

### Interpretation

Our comparison of mortality for people living with dementia depending on whether they had self-harmed is notable in that the pattern of results reverses depending on the strictness (or lack) of matching for age at first dementia diagnosis, particularly as our models all control for age as the timeline variable. We present these analyses openly to allow readers to assess the findings for themselves, but caution strong interpretation of the findings in either direction.

Previous research has shown all-cause mortality for people who self-harm is higher than the general population (Bergen et al., 2012), and our previous research showed those living with dementia who self-harmed tended to have more complex mental and physical health profiles than those who did not (Walker et al., 2023). It is less clear why mortality would be higher for people living with dementia who did not self-harm. People with moderate to severe dementia may be less able to plan and orchestrate self-harm, even if they had the intention to do so (Draper, 2015). This reduced ability to orchestrate self-harm would lead to fewer patients with severe dementia in the dementia and self-harm subgroup. This lowered number of people with severe dementia in the cohort may also emerge due to *diagnostic overshadowing* (Jones et al., 2008; Reiss et al., 1982). That is, the presence of severe dementia may make it difficult to determine if a given injury was self-inflicted or accidental. In both the above cases, fewer people with severe dementia would enter the dementia and self-harm subgroup. However, the evidence that dementia symptom severity is associated with an increased risk of mortality is mixed (Todd et al., 2013), so that, it is unclear if a bias against including people with severe dementia into the subgroup altered our results.

Another possibility is that those living with dementia who self-harmed may be more likely to receive appropriate health care, as the self-harm episode brings them into contact with health services. This extra support may lead to an increased lifespan for those living with dementia who self-harmed. This explanation raises the possibility that post-dementia diagnosis supports are currently inadequate (a view also reported by our lived experience advisory group), unless followed by an additional health crisis like self-harm that allows people to be connected with appropriate health services.

Notably, a higher number of mental health ambulatory days were associated with decreased mortality, with every 10 mental health ambulatory days in the previous year associated with a 9% drop in the rate of mortality. Previous mental health ambulatory days were also associated with an 8% decrease in repeat self-harm for every 10 days. Although we have shown that mental health ambulatory support decreased the risk of repeat self-harm, suicide in our cohort accounted for only 10 of the 456 deaths with a cause recorded (around 2.2%). That is, our results suggest that increased ambulatory mental health support may decrease not only the risk of future self-harm and suicide, but also all-cause mortality. This finding supports previous Australian research showing that receiving community mental health support substantially reduces all-cause mortality in older adults (Sharwood et al., 2023). In Australia, Older Persons’ Mental Health services provide aftercare for most older adults following self-harm alongside primary care (with the others being either referred back to their general practitioner, or not referred to any mental health services, Wand et al., 2023). The Older Persons’ Mental Health services also see people living with dementia when they have mental health comorbidity, but generally not people with dementia alone. It is possible that this identified decrease in mortality may be due to outpatient mental health services providing a bridge to other functional and health supports. For example, Older Person’s Mental Health services facilitate individualised access to multidisciplinary health services for older adults with mental health concerns (New South Wales Health, 2022; Wand et al., 2023). Overall, our data provide evidence that there may be benefits in linking to mental health supports immediately post-dementia diagnosis for people with a history of self-harm, echoing feedback from our lived experience advisory group. It is noteworthy that in NSW, aged care services and services run through Dementia Australia are the primary sites for routine post-diagnostic support for dementia. Older individuals with mental health comorbidity are eligible for mental health ambulatory care, although referral to such services declines substantially with age (Sharwood et al., 2023). However, as we do not have information on indication for ambulatory care (and cannot be sure that this finding does not represent a likelihood for less severe cases to be referred as priority to outpatient care), we caution against a strong interpretation of this finding.

### Strengths and limitations

Our study benefitted from a large population capture in NSW over a long time-period with reliable linkage (estimated 0.5% false positives, Reppermund et al., 2019), as well as the engagement of a lived experience dementia advocate group to help guide the planning of our study and interpretation of our results. It was limited by incomplete capture of health services (e.g. only hospital services and not primary health services), which means we do not have full, timely capture of all dementia and self-harm diagnoses. We also had incomplete capture of causes of death. Recent research from NSW has suggested that dementia is under-captured in hospital and deaths data, with a sensitivity of only around 20% compared to clinical assessment (although with a high specificity of 99%) (Chow et al., 2022). Furthermore, as noted above, there may be difficulties accurately identifying self-harm for people living with dementia. Also, due to the nature of our data, it is not possible to tell whether an individual who self-harmed intended suicide or self-harmed for another reason (such as feelings of hopelessness or loneliness, Wand et al., 2018). Finally, due to sample size we have grouped those who self-harm and then develop dementia with those who develop dementia and then go onto self-harm into one cohort for analysis. Our previous research indicates that the profile of these two groups is generally similar in age, with the time between first and second diagnoses usually within 2 years for both groups. However, those investigating the intersection of dementia and self-harm with larger samples may choose to examine the two groups separately.

## Conclusion

We investigated patterns and causes of mortality for people with dementia and self-harm diagnoses. We found that mortality increases when people who self-harm develop dementia, but could not strongly conclude whether self-harm increases or decreases mortality for people living with dementia. Promisingly, our results showed more ambulatory mental health use predicted a decrease in both mortality and repeat self-harm for people living with dementia who self-harmed. As such, we recommend a renewed focus on linking people with a prior diagnosis of self-harm to mental health services immediately after a dementia diagnosis.

## Supplemental Material

sj-docx-1-anp-10.1177_00048674241278243 – Supplemental material for Mortality in people living with dementia who self-harmed: An Australian data linkage study Supplemental material, sj-docx-1-anp-10.1177_00048674241278243 for Mortality in people living with dementia who self-harmed: An Australian data linkage study by Adrian R Walker, Preeyaporn Srasuebkul, Julian N Trollor, Anne PF Wand, Brian Draper, Rachael C Cvejic, Annette Moxey and Simone Reppermund in Australian & New Zealand Journal of Psychiatry
