# Supplementary material for: Mortality in people living with dementia who self-harmed: An Australian data linkage study
Source: Aust N Z J Psychiatry. 2024 Sep 9;58(11):990–1000. doi: 10.1177/00048674241278243 (PMC11497739; doi:10.1177/00048674241278243)
Supplement: sj-docx-1-anp-10.1177_00048674241278243 – Supplemental material for Mortality in people living with dementia who self-harmed: An Australian data linkage study [file sj-docx-1-anp-10.1177_00048674241278243.docx]

SUPPLEMENTARY - **Risk factors for mortality in people with dementia and self-harm diagnoses: an Australian data linkage study**

Walker, Adrian R.^1, 2^; Srasuebkul, Preeyaporn^3^; Trollor, Julian N. ^3,4^; Wand, Anne P.F.^5, 6, 7^; Draper, Brian^4, 6, 8^; Cvejic, Rachael C. ^1^; Moxey, Annette^9^; Reppermund, Simone^1, 4.^

^1^ The Department of Developmental Disability Neuropsychiatry, Faculty of Medicine and Health, UNSW Sydney, NSW 2052, Australia (Institute of origin for this manuscript)

^2^ Centre for Big Data in Health, Faculty of Medicine and Health, UNSW Sydney, NSW 2052, Australia

^3^ National Centre of Excellence in Intellectual Disability Health, Faculty of Medicine and Health, UNSW Sydney, NSW 2052, Australia

^4^ Centre for Healthy Brain Ageing, Faculty of Medicine and Health, UNSW Sydney, NSW 2052, Australia

^5^ Speciality of Psychiatry, Faculty of Medicine and Health, University of Sydney, Camperdown, NSW 2006, Australia

^6^ Discipline of Psychiatry and Mental Health, Faculty of Medicine and Health, UNSW Sydney, NSW 2052, Australia

^7^ Older Peoples Mental Health Service, Sydney Local Health District, c/o Concord Centre for Mental Health, Concord, NSW, 2139.

^8^ Eastern Suburbs Older Persons Mental Health Service, Prince of Wales Hospital, Randwick, NSW 2031, Australia

^9^ Dementia Australia, Endeavour House, 2-10 Captain Cook Crescent, Griffith, ACT 2603, Australia

# Sensitivity analysis excluding mild cognitive impairment as an indicator of dementia

## Cohort demographic summary

**Table S1: Rates of death per 1,000 person years (without mild cognitive impairment as an indicator of dementia)**

| **Cohort or subgroup** | **Rate of death per 1,000 person years** |
| --- | --- |
| Dementia cohort* | 239.0 |
| Self-harm cohort* | 20.4 |
| Dementia and self-harm subgroup | 136.6 |
| *Excluding person time for those in the dementia and self-harm subgroup | |

## Figure S1: Hazard ratios for death, with those in the dementia and self-harm subgroup as the baseline. Error bars represent 95% confidence intervals

**(without mild cognitive impairment as an indicator of dementia). Note that due to failure to converge the dementia cohort model only had one spline**


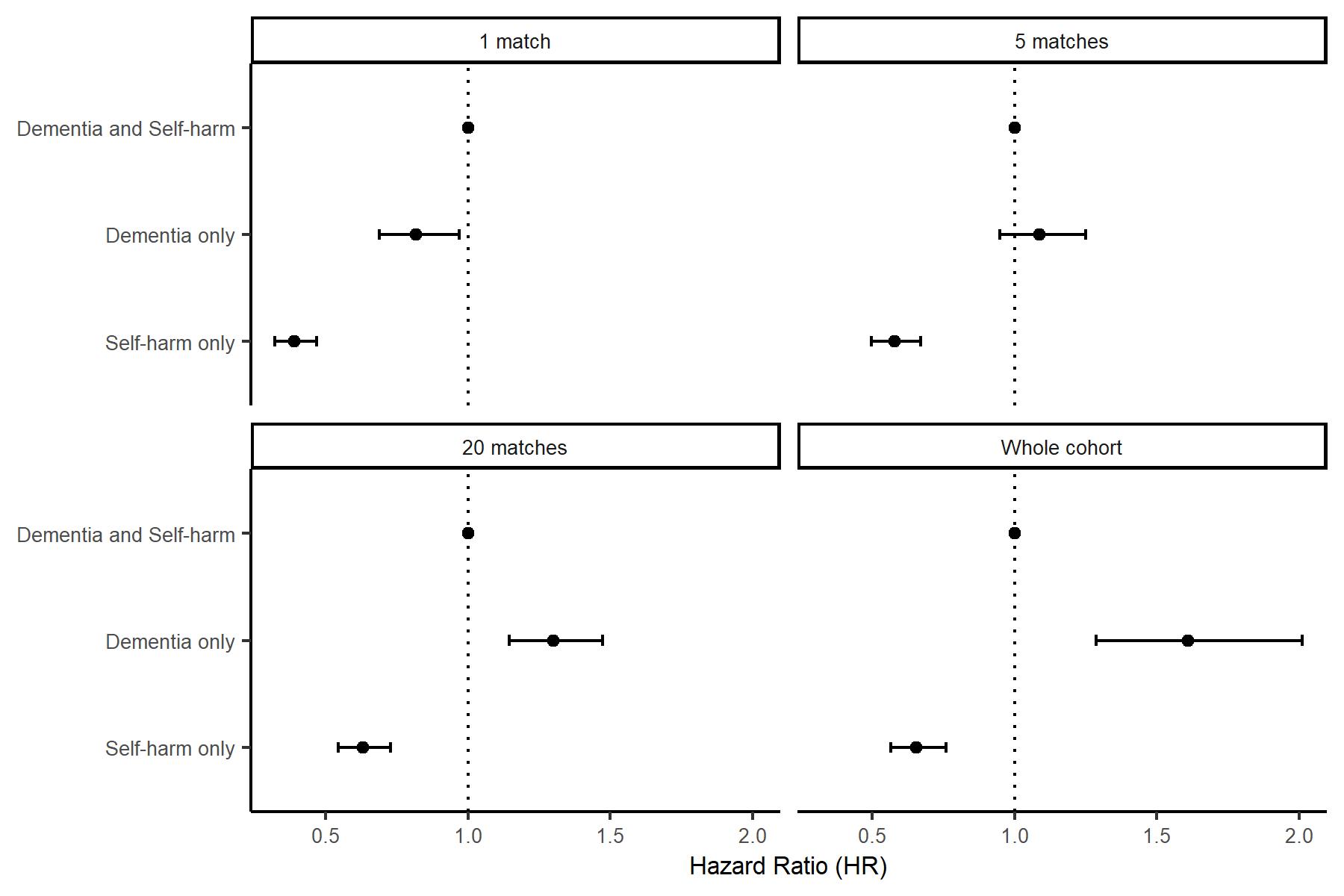


## Dementia and self-harm subgroup

## Figure S2: Density of time to death after entry into the dementia and self-harm subgroup (without mild cognitive impairment as an indicator of dementia)


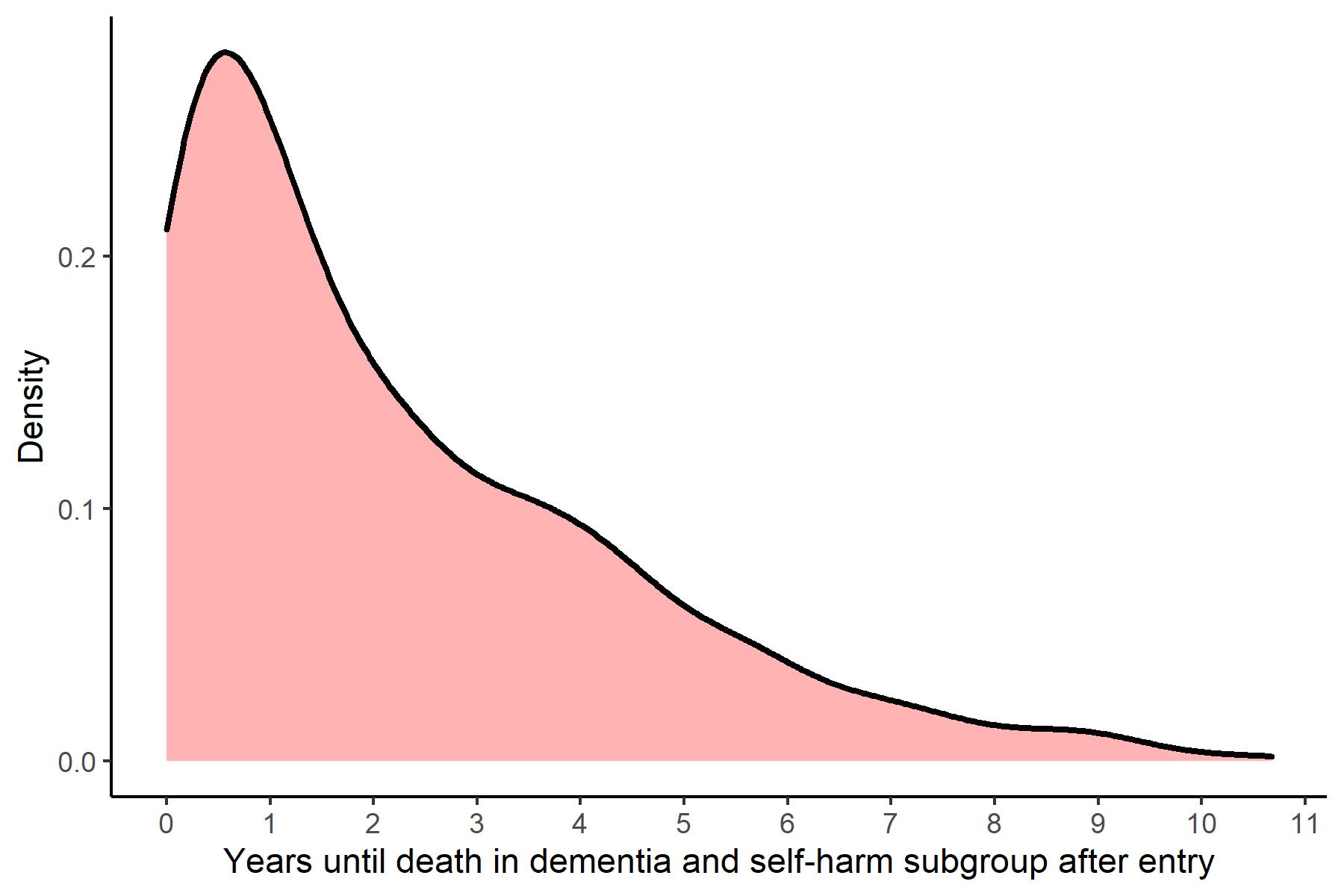


## Table S2: Causes of death by International Classification of Diseases – 10^th^ Revision (ICD10) until 31^st^ of December 2013 (without mild cognitive impairment as an indicator of dementia).

| **International Classification of Diseases – 10^th^ Revision (ICD10) Chapter** | **Dementia cohort*** | **Self-harm cohort*** | **Dementia and self-harm subgroup** |
| --- | --- | --- | --- |
| **Total** | **90,422** | **2236** | **443** |
| 1. Certain infectious and parasitic diseases | 1601 (1.8%) | 54 (2.4%) | 16 (3.6%) |
| 2. Neoplasms | 9643 (10.7%) | >432 (19.3%) | 67 (15.1%) |
| 3. Blood and blood forming organs | 218 (0.2%) | ** | 0 |
| 4. Endocrine | 3797 (4.2%) | >62 (2.8%) | <20 (4.5%) |
| 5. Mental and behavioural | 13,710 (15.2%) | >53 (2.4%) | <44 (9.9%) |
| 6. Nervous | 8325 (9.2%) | 57 (2.5%) | 39 (8.8%) |
| 7. Eye and Adnexa | ** | 0 | 0 |
| 8. Ear and Mastoid | ** | 0 | 0 |
| 9. Circulatory | 35,798 (39.6%) | >448 (20.0%) | 146 (33.0%) |
| 10. Respiratory | 7845 (8.7%) | >167 (7.5%) | <43 (9.7%) |
| 11. Digestive | 2875 (3.2%) | 124 (5.5%) | 32 (7.2%) |
| 12. Skin and subcutaneous | 390 (0.4%) | * | 0 |
| 13. Musculoskeletal | 630 (0.7%) | 12 (0.5%) | ** |
| 14. Genitourinary | 2625 (2.9%) | 26 (1.2)% | 9 (2.0%) |
| 17. Congenital | <99 (0.1%) | * | 0 |
| 18. Signs and symptoms | 609 (0.7%) | 23 (1.0%) | ** |
| 20. External causes (self-harm) | ** | >409 (18.3%) | <10 (2.3%) |
| 20. External causes (not self-harm) | 2248 (2.5%) | >344 (15.4%) | 25 (5.6%) |
| *Excludes those who entered the dementia and self-harm subgroup |  |  |  |
| **Data censored due to small cell size |  |  |  |
| Note: Some cells only indicate over or under the value in the main manuscript Table 2. This is due the change in cell size being too small between the two tables, and risking deanonymisation. | | | |

## Table S3: Predictors of death for people in the dementia and self-harm subgroup with age as the time scale

**(without mild cognitive impairment as an indicator of dementia)**

|  | **Variables individually adjusted according to causal model** | | | **All variables included model** | | |
| --- | --- | --- | --- | --- | --- | --- |
| **Variable** | **Incident Rate Ratio (95% CI)** | **Standard Error** | **p value** | **Incident Rate Ratio (95% CI)** | **Standard Error** | **p value** |
| **Sex (Female)**^✝^ | 0.69 (0.59, 0.8) | 0.06 | <0.001 | 0.73 (0.62, 0.86) | 0.06 | <0.001 |
|  |  |  |  |  |  |  |
| **Remoteness**^✝^ |  |  | 0.512* |  |  | 0.035* |
| Major cities (Reference) | 1 | … | … | 1 | … | … |
| Inner Regional | 0.95 (0.78, 1.15) | 0.09 | 0.573 | 0.99 (0.8, 1.23) | 0.11 | 0.923 |
| Outer regional and beyond | 1.18 (0.83, 1.67) | 0.21 | 0.35 | 1.56 (1.1, 2.21) | 0.28 | 0.012 |
|  |  |  |  |  |  |  |
| **Index of relative socioeconomic disadvantage quintile**^✝^ |  |  | 0.717* |  |  | 0.690* |
| 1-2 (Most disadvantaged) | 1 | … | … | 1 | … | … |
| 3-4 | 0.92 (0.74, 1.14) | 0.1 | 0.452 | 0.9 (0.73, 1.12) | 0.1 | 0.342 |
| 5-6 | 0.87 (0.69, 1.1) | 0.1 | 0.241 | 0.86 (0.67, 1.11) | 0.11 | 0.249 |
| 7-8 | 0.97 (0.75, 1.25) | 0.12 | 0.802 | 0.97 (0.75, 1.26) | 0.13 | 0.82 |
| 9-10 (Least disadvantaged) | 1.02 (0.81, 1.28) | 0.12 | 0.894 | 1 (0.79, 1.28) | 0.12 | 0.976 |
|  |  |  |  |  |  |  |
| **Marital Status**^✝^ |  |  | <0.001* |  |  | <0.001* |
| Married/De Facto | 1 | … | … | 1 | … | … |
| Divorced/Widowed/Separated | 0.93 (0.8, 1.1) | 0.08 | 0.406 | 0.93 (0.79, 1.09) | 0.08 | 0.358 |
| Never Married | 1.1 (0.82, 1.48) | 0.16 | 0.526 | 1.08 (0.8, 1.46) | 0.16 | 0.598 |
|  |  |  |  |  |  |  |
| **Num. Elixhauser comorbidities in year prior** | 1.22 (1.18, 1.27) | 0.02 | <0.001 | 1.18 (1.14, 1.23) | 0.02 | <0.001 |
|  |  |  |  |  |  |  |
| **Mental health Ambulatory use in year prior (per 10 days)** | 0.92 (0.85, 0.99) | 0.04 | 0.031 | 0.91 (0.83, 0.98) | 0.04 | 0.02 |
|  |  |  |  |  |  |  |
| **Involuntary mental health admissions in year prior (per 10 admissions)** | 1.01 (0.98, 1.03) | 0.01 | 0.556 | 1.01 (0.99, 1.03) | 0.01 | 0.424 |
|  |  |  |  |  |  |  |
| **Emergency department presentations in year prior  (per 10 presentations)^$^** | 1.15 (1, 1.31) | 0.08 | 0.043 | 1.17 (1.02, 1.34) | 0.08 | 0.022 |
|  |  |  |  |  |  |  |
| **History of depression** | 0.98 (0.84, 1.16) | 0.08 | 0.841 | 0.99 (0.83, 1.18) | 0.09 | 0.919 |
|  |  |  |  |  |  |  |
| **History of drug or alcohol abuse** | 1.21 (1.02, 1.45) | 0.11 | 0.034 | 1.19 (0.98, 1.44) | 0.12 | 0.078 |
|  |  |  |  |  |  |  |
| **History of psychotic disorder** | 1.08 (0.91, 1.28) | 0.09 | 0.368 | 1.04 (0.86, 1.25) | 0.1 | 0.691 |
|  |  |  |  |  |  |  |
| **History of anxiety disorder** | 0.96 (0.82, 1.12) | 0.08 | 0.58 | 0.93 (0.79, 1.1) | 0.08 | 0.422 |
|  |  |  |  |  |  |  |
| **History of delirium** | 1.57 (1.32, 1.87) | 0.14 | <0.001 | 1.57 (1.32, 1.87) | 0.14 | <0.001 |
|  |  |  |  |  |  |  |
| **History of behavioural problems** | 1.17 (1, 1.38) | 0.1 | 0.052 | 1.14 (0.95, 1.37) | 0.11 | 0.158 |
|  |  |  |  |  |  |  |
| **History of personality disorders** | 0.87 (0.69, 1.1) | 0.1 | 0.255 | 0.8 (0.62, 1.03) | 0.1 | 0.087 |
| * Wald test for variable inclusion | | | | | | |
| ^✝^ “Unknown” category excluded from output | | | | | | |
| ^$^ To allow for model convergence, if someone had over 100 presentations to ED in a single year, we treated them as having at most 101 presentations in that year. | | | | | | |

## Table S4: Survival analysis results for comparison of rate of death between cohorts (1-nearest matching without mild cognitive impairment as an indicator of dementia)

|  | **Dementia cohort** | | | **Self-harm cohort** | | |
| --- | --- | --- | --- | --- | --- | --- |
| **Variable** | **Incident Rate Ratio (95% CI)** | **Standard Error** | **p value** | **Incident Rate Ratio (95% CI)** | **Standard Error** | **p value** |
| **Cohort/subgroup** |  |  |  |  |  |  |
| Dementia and Self-harm | 1 | … | … | 1 | … | … |
| Dementia only | 0.82 (0.69, 0.97) | 0.07 | 0.02 | … | … | … |
| Self-harm only | … | … | … | 0.39 (0.32, 0.47) | 0.04 | <0.001 |
|  |  |  |  |  |  |  |
| **Sex (Female)**^✝^ | 1 (1, 1) | <0.01 | <0.001 | 1 (1, 1) | <0.01 | 0.002 |
|  |  |  |  |  |  |  |
| **Remoteness**^✝^ |  |  |  |  |  |  |
| Major cities (Reference) | 1 | … | … | 1 | … | … |
| Inner Regional | 1.21 (0.99, 1.48) | 0.12 | 0.059 | 1.05 (0.83, 1.33) | 0.13 | 0.692 |
| Outer regional and beyond | 1.49 (1.03, 2.13) | 0.27 | 0.032 | 1.45 (1.01, 2.08) | 0.27 | 0.044 |
|  |  |  |  |  |  |  |
| **Index of relative socioeconomic disadvantage quintile**^✝^ |  |  |  |  |  |  |
| 1-2 (Most disadvantaged) | 1 | … | … | 1 | … | … |
| 3-4 | 0.88 (0.71, 1.09) | 0.1 | 0.243 | 0.96 (0.76, 1.23) | 0.12 | 0.77 |
| 5-6 | 1.06 (0.83, 1.36) | 0.13 | 0.621 | 0.88 (0.66, 1.17) | 0.13 | 0.381 |
| 7-8 | 0.96 (0.74, 1.26) | 0.13 | 0.792 | 0.96 (0.71, 1.29) | 0.15 | 0.776 |
| 9-10 (Least disadvantaged) | 0.92 (0.72, 1.17) | 0.11 | 0.493 | 0.97 (0.74, 1.29) | 0.14 | 0.859 |
|  |  |  |  |  |  |  |
| **Marital Status**^✝^ |  |  |  |  |  |  |
| Married/De Facto | 1 | … | … | 1 | … | … |
| Divorced/Widowed/Separated | 0.88 (0.74, 1.05) | 0.08 | 0.156 | 0.9 (0.75, 1.09) | 0.09 | 0.284 |
| Never Married | 1.14 (0.87, 1.49) | 0.16 | 0.348 | 1.26 (0.91, 1.73) | 0.21 | 0.161 |
|  |  |  |  |  |  |  |
| **Num. Elixhauser comorbidities in year prior** | 1.2 (1.15, 1.26) | 0.03 | <0.001 | 1.22 (1.17, 1.28) | 0.03 | <0.001 |
|  |  |  |  |  |  |  |
| **Mental health Ambulatory use in year prior (per 10 days)** | 0.9 (0.8, 1) | 0.05 | 0.059 | 0.83 (0.73, 0.93) | 0.05 | 0.002 |
|  |  |  |  |  |  |  |
| **Involuntary mental health admissions in year prior (per 10 admissions)** | 0.98 (0.94, 1.03) | 0.02 | 0.523 | 1.04 (0.97, 1.11) | 0.03 | 0.275 |
|  |  |  |  |  |  |  |
| **Emergency department presentations in year prior (per 10 presentations)^$^** | 1.25 (1.05, 1.48) | 0.11 | 0.011 | 1.16 (0.99, 1.36) | 0.09 | 0.075 |
|  |  |  |  |  |  |  |
| **History of depression** | 0.84 (0.7, 1) | 0.08 | 0.056 | 0.92 (0.76, 1.12) | 0.09 | 0.403 |
|  |  |  |  |  |  |  |
| **History of drug or alcohol abuse** | 0.98 (0.8, 1.2) | 0.1 | 0.812 | 1.39 (1.11, 1.73) | 0.16 | 0.004 |
|  |  |  |  |  |  |  |
| **History of psychotic disorder** | 0.84 (0.69, 1.03) | 0.09 | 0.09 | 0.9 (0.72, 1.12) | 0.1 | 0.339 |
|  |  |  |  |  |  |  |
| **History of anxiety disorder** | 0.77 (0.63, 0.95) | 0.08 | 0.013 | 1.04 (0.87, 1.25) | 0.1 | 0.651 |
|  |  |  |  |  |  |  |
| **History of delirium** | 1.35 (1.11, 1.64) | 0.13 | 0.003 | 1.51 (1.23, 1.84) | 0.15 | <0.001 |
|  |  |  |  |  |  |  |
| **History of behavioural problems** | 1.05 (0.85, 1.3) | 0.11 | 0.639 | 1.05 (0.85, 1.29) | 0.11 | 0.654 |
|  |  |  |  |  |  |  |
| **History of personality disorders** | 0.72 (0.52, 1.01) | 0.12 | 0.057 | 0.75 (0.56, 1.02) | 0.11 | 0.063 |
| ^✝^ “Unknown” category excluded from output | | | | | | |
| ^$^ To allow for model convergence, if someone had over 100 presentations to ED in a single year, we treated them as having at most 101 presentations in that year. | | | | | | |

Figure S3: Comparison of age at first presentation for dementia (left) and self-harm (right) for 1-nearest matching analysis  **(without mild cognitive impairment as an indicator of dementia)**


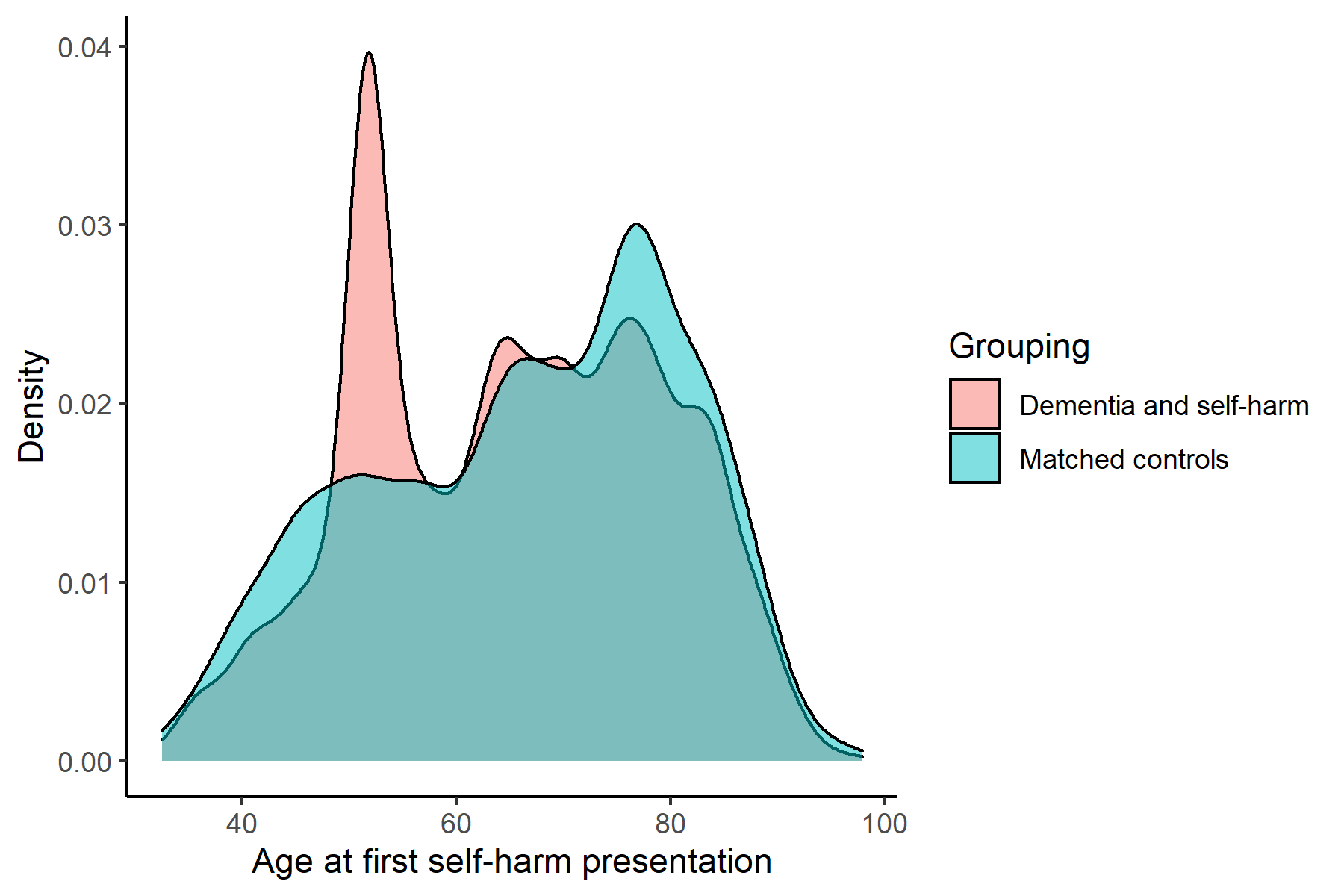

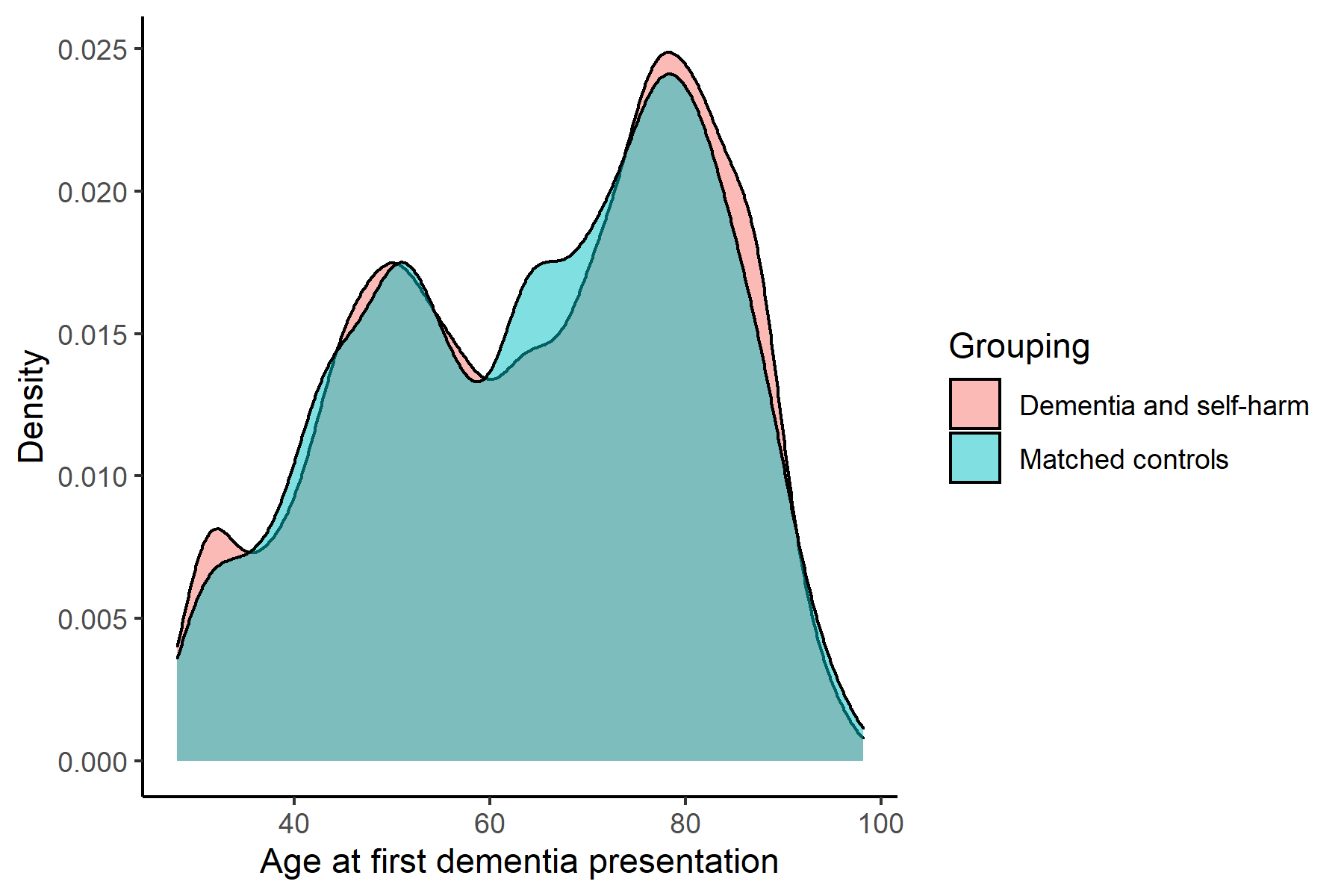


## Table S5: Survival analysis results for comparison of rate of death between cohorts (5-nearest matching without mild cognitive impairment as an indicator of dementia)

|  | **Dementia cohort** | | | **Self-harm cohort** | | |
| --- | --- | --- | --- | --- | --- | --- |
| **Variable** | **Incident Rate Ratio (95% CI)** | **Standard Error** | **p value** | **Incident Rate Ratio (95% CI)** | **Standard Error** | **p value** |
| **Cohort/subgroup** |  |  |  |  |  |  |
| Dementia and Self-harm | 1 | … | … | 1 | … | … |
| Dementia only | 1.09 (0.95, 1.25) | 0.08 | 0.227 | … | … | … |
| Self-harm only | … | … | … | 0.58 (0.5, 0.67) | 0.04 | <0.001 |
|  |  |  |  |  |  |  |
| **Sex (Female)**^✝^ | 1 (1, 1) | <0.01 | <0.001 | 1 (1, 1) | <0.01 | <0.001 |
|  |  |  |  |  |  |  |
| **Remoteness**^✝^ |  |  |  |  |  |  |
| Major cities (Reference) | 1 | … | … | 1 | … | … |
| Inner Regional | 1.07 (0.95, 1.2) | 0.06 | 0.259 | 1.03 (0.89, 1.19) | 0.08 | 0.731 |
| Outer regional and beyond | 1.14 (0.95, 1.37) | 0.11 | 0.165 | 1.16 (0.92, 1.46) | 0.13 | 0.207 |
|  |  |  |  |  |  |  |
| **Index of relative socioeconomic disadvantage quintile**^✝^ |  |  |  |  |  |  |
| 1-2 (Most disadvantaged) | 1 | … | … | 1 | … | … |
| 3-4 | 0.85 (0.75, 0.96) | 0.05 | 0.007 | 1 (0.85, 1.16) | 0.08 | 0.964 |
| 5-6 | 0.95 (0.83, 1.09) | 0.07 | 0.47 | 0.96 (0.8, 1.15) | 0.09 | 0.633 |
| 7-8 | 0.88 (0.76, 1.03) | 0.07 | 0.102 | 0.86 (0.7, 1.05) | 0.09 | 0.136 |
| 9-10 (Least disadvantaged) | 0.94 (0.81, 1.08) | 0.07 | 0.357 | 0.91 (0.75, 1.1) | 0.09 | 0.332 |
|  |  |  |  |  |  |  |
| **Marital Status**^✝^ |  |  |  |  |  |  |
| Married/De Facto | 1 | … | … | 1 | … | … |
| Divorced/Widowed/Separated | 0.94 (0.85, 1.03) | 0.05 | 0.184 | 1.01 (0.9, 1.14) | 0.06 | 0.817 |
| Never Married | 1.07 (0.91, 1.25) | 0.09 | 0.395 | 1.11 (0.89, 1.39) | 0.13 | 0.362 |
|  |  |  |  |  |  |  |
| **Num. Elixhauser comorbidities in year prior** | 1.17 (1.14, 1.2) | 0.01 | <0.001 | 1.3 (1.26, 1.34) | 0.02 | <0.001 |
|  |  |  |  |  |  |  |
| **Mental health Ambulatory use in year prior (per 10 days)** | 0.84 (0.76, 0.94) | 0.05 | 0.002 | 0.9 (0.84, 0.97) | 0.03 | 0.003 |
|  |  |  |  |  |  |  |
| **Involuntary mental health admissions in year prior (per 10 admissions)** | 1 (0.99, 1.01) | 0.01 | 0.65 | 1.02 (0.96, 1.08) | 0.03 | 0.521 |
|  |  |  |  |  |  |  |
| **Emergency department presentations in year prior (per 10 presentations)^$^** | 1.3 (1.13, 1.5) | 0.09 | <0.001 | 1.27 (1.12, 1.43) | 0.08 | <0.001 |
|  |  |  |  |  |  |  |
| **History of depression** | 0.89 (0.79, 0.99) | 0.05 | 0.039 | 1 (0.89, 1.14) | 0.06 | 0.946 |
|  |  |  |  |  |  |  |
| **History of drug or alcohol abuse** | 1.05 (0.93, 1.19) | 0.07 | 0.415 | 1.15 (1.01, 1.31) | 0.08 | 0.039 |
|  |  |  |  |  |  |  |
| **History of psychotic disorder** | 0.79 (0.7, 0.9) | 0.05 | <0.001 | 0.9 (0.77, 1.06) | 0.07 | 0.202 |
|  |  |  |  |  |  |  |
| **History of anxiety disorder** | 0.79 (0.69, 0.89) | 0.05 | <0.001 | 0.99 (0.88, 1.11) | 0.06 | 0.874 |
|  |  |  |  |  |  |  |
| **History of delirium** | 1.35 (1.21, 1.5) | 0.07 | <0.001 | 1.57 (1.35, 1.82) | 0.12 | <0.001 |
|  |  |  |  |  |  |  |
| **History of behavioural problems** | 1.26 (1.11, 1.44) | 0.08 | <0.001 | 1.06 (0.93, 1.21) | 0.07 | 0.356 |
|  |  |  |  |  |  |  |
| **History of personality disorders** | 0.82 (0.65, 1.03) | 0.1 | 0.082 | 0.75 (0.61, 0.93) | 0.08 | 0.009 |
| ^✝^ “Unknown” category excluded from output | | | | | | |
| ^$^ To allow for model convergence, if someone had over 100 presentations to ED in a single year, we treated them as having at most 101 presentations in that year. | | | | | | |

**Figure S4: Comparison of age at first presentation for dementia (left) and self-harm (right) for 5-nearest matching analysis (without mild cognitive impairment as an indicator of dementia)**


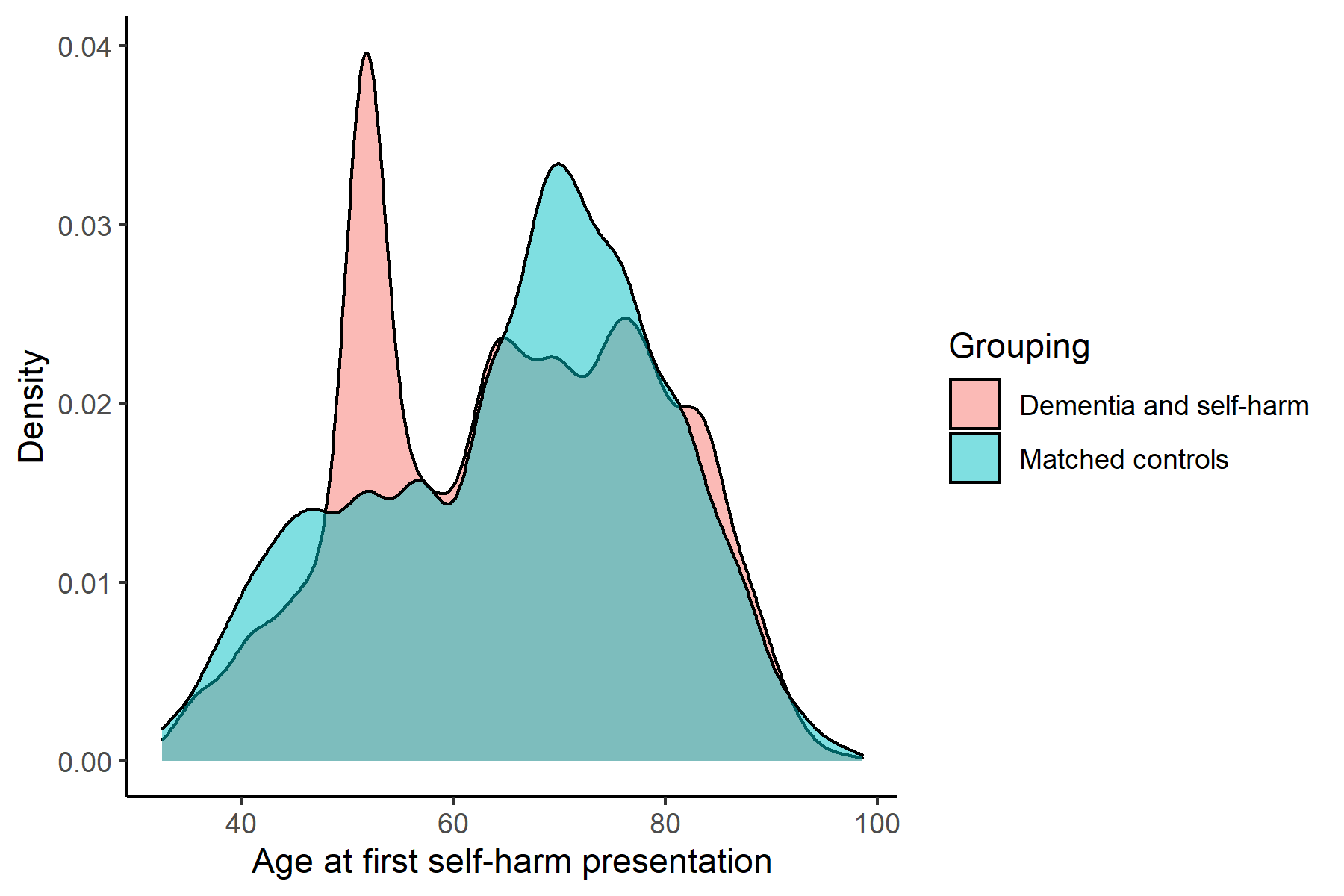

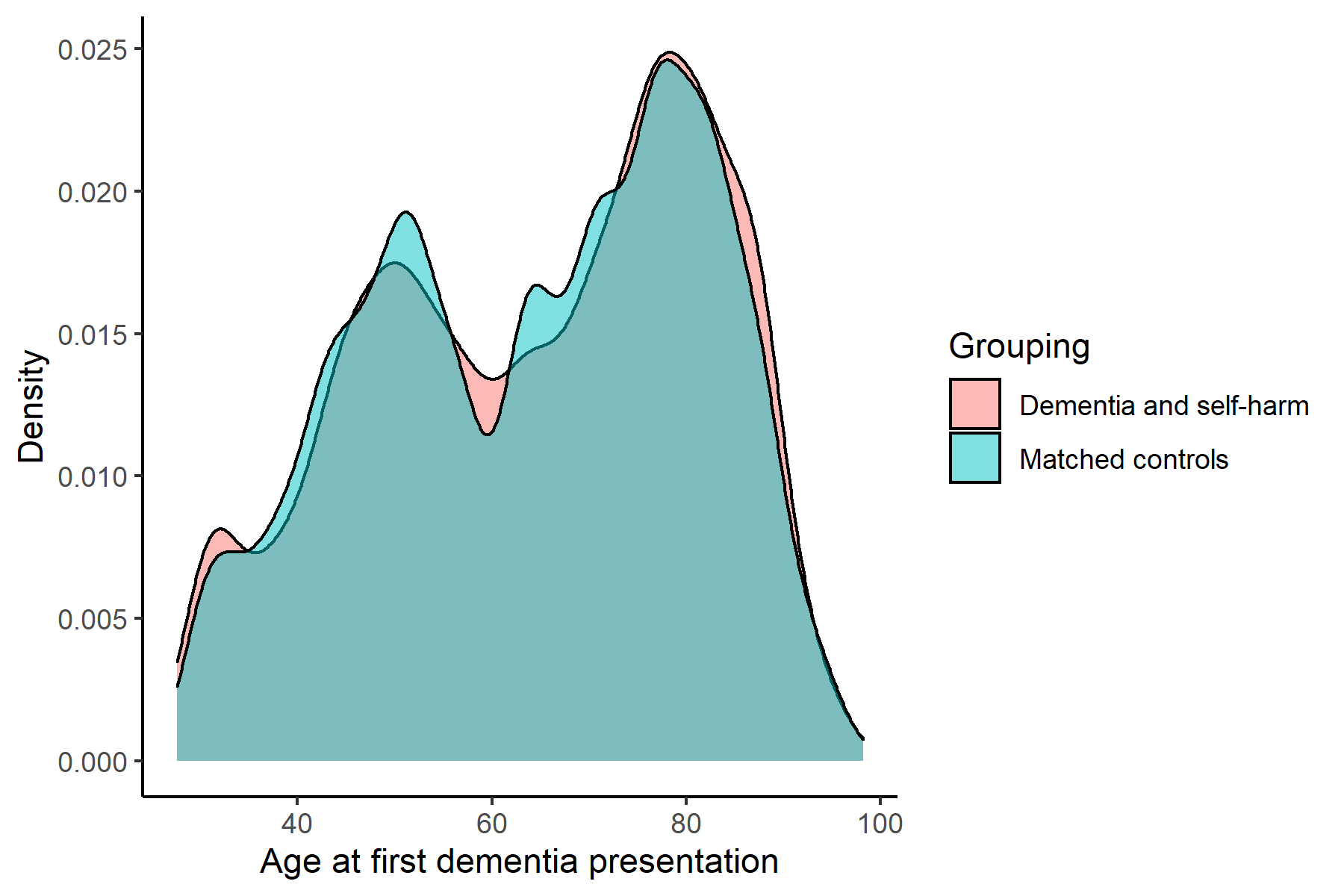


## Table S6: Survival analysis results for comparison of rate of death between cohorts (20-nearest matching without mild cognitive impairment as an indicator of dementia).

|  | **Dementia cohort** | | | **Self-harm cohort** | | |
| --- | --- | --- | --- | --- | --- | --- |
| **Variable** | **Incident Rate Ratio (95% CI)** | **Standard Error** | **p value** | **Incident Rate Ratio (95% CI)** | **Standard Error** | **p value** |
| **Cohort/subgroup** |  |  |  |  |  |  |
| Dementia and Self-harm | 1 | … | … | 1 | … | … |
| Dementia only | 1.3 (1.14, 1.47) | <0.01 | 0.002 | … | … | … |
| Self-harm only | … | … | … | 0.63 (0.54, 0.73) | 0.05 | <0.001 |
|  |  |  |  |  |  |  |
| **Sex (Female)**^✝^ | 0.82 (0.77, 0.86) | 0.02 | <0.001 | 1 (1, 1) | <0.01 | 0.003 |
|  |  |  |  |  |  |  |
| **Remoteness**^✝^ |  |  |  |  |  |  |
| Major cities (Reference) | 1 | … | … | 1 | … | … |
| Inner Regional | 1.08 (1.02, 1.15) | 0.03 | 0.011 | 1.09 (0.98, 1.21) | 0.06 | 0.107 |
| Outer regional and beyond | 1.03 (0.94, 1.14) | 0.05 | 0.532 | 1.07 (0.91, 1.27) | 0.09 | 0.402 |
|  |  |  |  |  |  |  |
| **Index of relative socioeconomic disadvantage quintile**^✝^ |  |  |  |  |  |  |
| 1-2 (Most disadvantaged) | 1 | … | … | 1 | … | … |
| 3-4 | 0.95 (0.89, 1.02) | 0.03 | 0.133 | 1.02 (0.91, 1.14) | 0.06 | 0.727 |
| 5-6 | 0.98 (0.92, 1.06) | 0.04 | 0.678 | 1.01 (0.88, 1.15) | 0.07 | 0.93 |
| 7-8 | 0.92 (0.85, 1) | 0.04 | 0.057 | 0.89 (0.77, 1.04) | 0.07 | 0.147 |
| 9-10 (Least disadvantaged) | 0.94 (0.87, 1.01) | 0.04 | 0.084 | 0.98 (0.85, 1.14) | 0.07 | 0.803 |
|  |  |  |  |  |  |  |
| **Marital Status**^✝^ |  |  |  |  |  |  |
| Married/De Facto | 1 | … | … | 1 | … | … |
| Divorced/Widowed/Separated | 1.01 (0.96, 1.06) | 0.03 | 0.784 | 1.13 (1.03, 1.23) | 0.05 | 0.01 |
| Never Married | 1.11 (1.02, 1.2) | 0.05 | 0.016 | 1.31 (1.15, 1.5) | 0.09 | <0.001 |
|  |  |  |  |  |  |  |
| **Num. Elixhauser comorbidities in year prior** | 1.17 (1.15, 1.18) | 0.01 | <0.001 | 1.34 (1.31, 1.38) | 0.02 | <0.001 |
|  |  |  |  |  |  |  |
| **Mental health Ambulatory use in year prior (per 10 days)** | 0.91 (0.85, 0.96) | 0.03 | 0.001 | 0.96 (0.93, 1) | 0.02 | 0.025 |
|  |  |  |  |  |  |  |
| **Involuntary mental health admissions in year prior (per 10 admissions)** | 0.99 (0.99, 1) | <0.01 | 0.162 | 1 (0.99, 1.01) | 0.01 | 0.962 |
|  |  |  |  |  |  |  |
| **Emergency department presentations in year prior (per 10 presentations)^$^** | 1.37 (1.25, 1.5) | 0.06 | <0.001 | 1.27 (1.19, 1.34) | 0.04 | <0.001 |
|  |  |  |  |  |  |  |
| **History of depression** | 0.95 (0.89, 1.01) | 0.03 | 0.089 | 1.02 (0.93, 1.11) | 0.05 | 0.726 |
|  |  |  |  |  |  |  |
| **History of drug or alcohol abuse** | 1.07 (1.01, 1.15) | 0.04 | 0.032 | 1.4 (1.28, 1.53) | 0.06 | <0.001 |
|  |  |  |  |  |  |  |
| **History of psychotic disorder** | 0.82 (0.77, 0.88) | 0.03 | <0.001 | 1.1 (0.99, 1.23) | 0.06 | 0.076 |
|  |  |  |  |  |  |  |
| **History of anxiety disorder** | 0.84 (0.78, 0.91) | 0.03 | <0.001 | 0.94 (0.86, 1.02) | 0.04 | 0.15 |
|  |  |  |  |  |  |  |
| **History of delirium** | 1.33 (1.25, 1.41) | 0.04 | <0.001 | 1.59 (1.39, 1.8) | 0.1 | <0.001 |
|  |  |  |  |  |  |  |
| **History of behavioural problems** | 1.26 (1.17, 1.35) | 0.05 | <0.001 | 1.1 (1, 1.2) | 0.05 | 0.055 |
|  |  |  |  |  |  |  |
| **History of personality disorders** | 0.91 (0.79, 1.04) | 0.06 | 0.154 | 0.84 (0.74, 0.96) | 0.06 | 0.009 |
| ^✝^ “Unknown” category excluded from output | | | | | | |
| ^$^ To allow for model convergence, if someone had over 100 presentations to ED in a single year, we treated them as having at most 101 presentations in that year. | | | | | | |

**Figure S5: Comparison of age at first presentation for dementia (left) and self-harm (right) for 20-nearest matching analysis (without mild cognitive impairment as an indicator of dementia)**


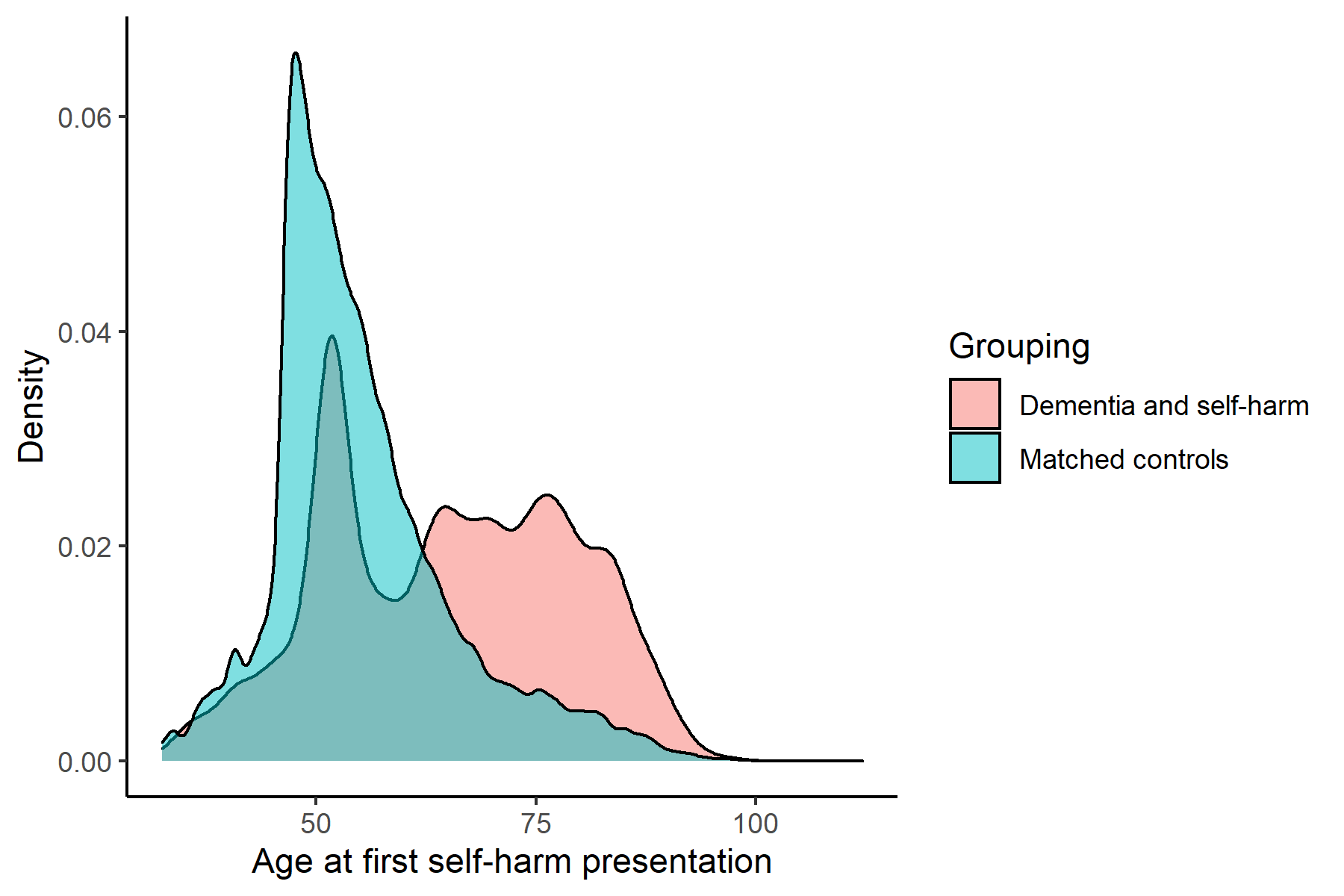

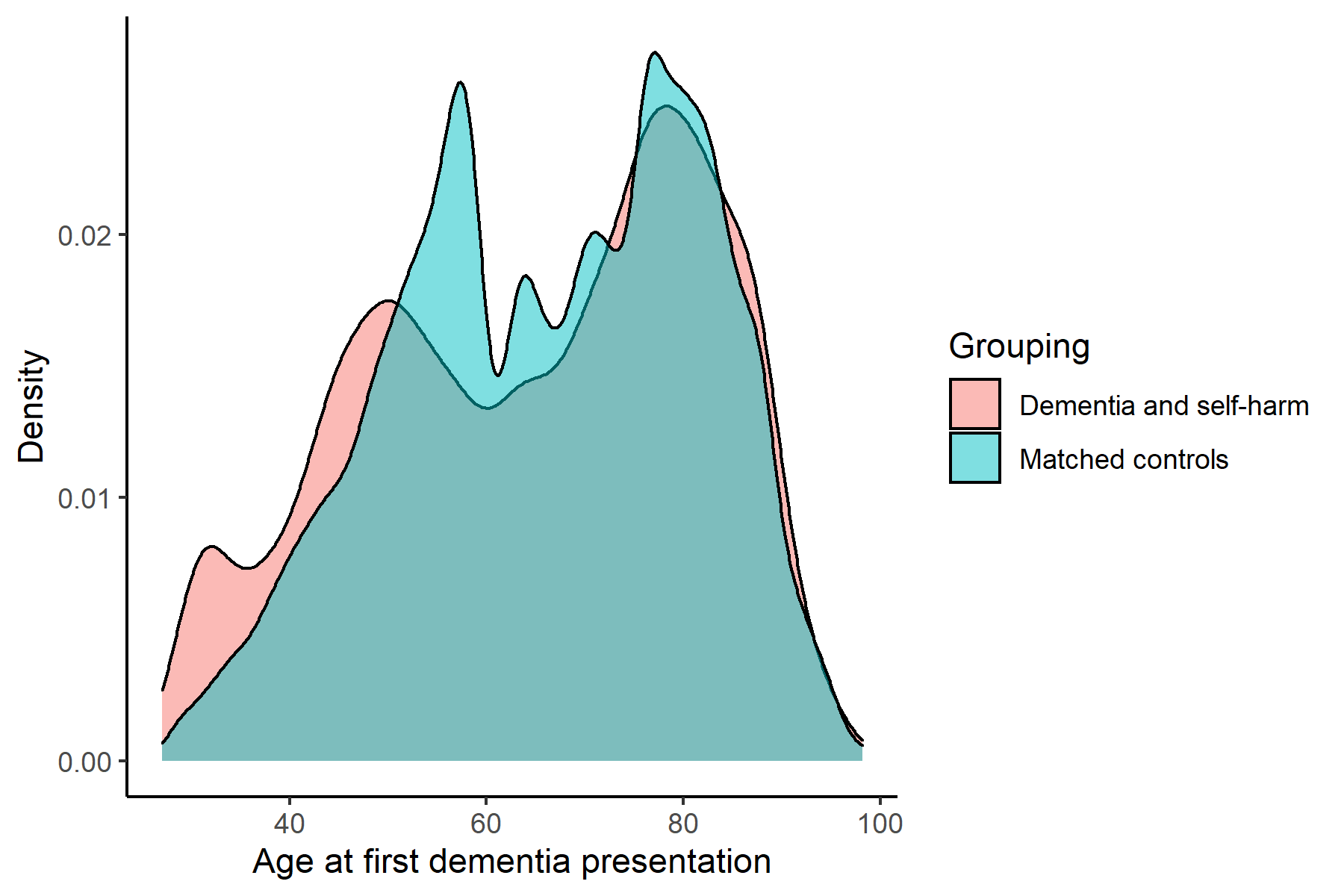


## Table S7: Survival analysis results for comparison of rate of death between cohorts (whole cohort without mild cognitive impairment as an indicator of dementia)

|  | **Dementia cohort** | | | **Self-harm cohort** | | |
| --- | --- | --- | --- | --- | --- | --- |
| **Variable** | **Incident Rate Ratio (95% CI)** | **Standard Error** | **p value** | **Incident Rate Ratio (95% CI)** | **Standard Error** | **p value** |
| **Cohort/subgroup** |  |  |  |  |  |  |
| Dementia and Self-harm | 1 | … | … | 1 | … | … |
| Dementia only | 1.61 (1.29, 2.01) | 0.18 | <0.001 | … | … | … |
| Self-harm only | … | … | … | 0.66 (0.57, 0.76) | 0.05 | <0.001 |
|  |  |  |  |  |  |  |
| **Sex (Female)**^✝^ | 0.75 (0.74, 0.76) | 0.01 | <0.001 | 1 (1, 1) | <0.01 | 0.61 |
|  |  |  |  |  |  |  |
| **Remoteness**^✝^ |  |  |  |  |  |  |
| Major cities (Reference) | 1 | … | … | 1 | … | … |
| Inner Regional | 1.09 (1.07, 1.11) | 0.01 | <0.001 | 1.06 (0.97, 1.16) | 0.05 | 0.202 |
| Outer regional and beyond | 1.07 (1.04, 1.11) | 0.02 | <0.001 | 1.02 (0.88, 1.19) | 0.08 | 0.768 |
|  |  |  |  |  |  |  |
| **Index of relative socioeconomic disadvantage quintile**^✝^ |  |  |  |  |  |  |
| 1-2 (Most disadvantaged) | 1 | … | … | 1 | … | … |
| 3-4 | 0.99 (0.97, 1) | 0.01 | 0.139 | 0.99 (0.9, 1.1) | 0.05 | 0.906 |
| 5-6 | 0.96 (0.94, 0.98) | 0.01 | <0.001 | 0.97 (0.87, 1.1) | 0.06 | 0.668 |
| 7-8 | 0.97 (0.95, 0.99) | 0.01 | 0.011 | 0.87 (0.76, 1) | 0.06 | 0.049 |
| 9-10 (Least disadvantaged) | 0.91 (0.89, 0.93) | 0.01 | <0.001 | 0.95 (0.83, 1.08) | 0.06 | 0.442 |
|  |  |  |  |  |  |  |
| **Marital Status**^✝^ |  |  |  |  |  |  |
| Married/De Facto | 1 | … | … | 1 | … | … |
| Divorced/Widowed/Separated | 1.02 (1, 1.03) | 0.01 | 0.029 | 1.16 (1.07, 1.26) | 0.05 | 0.001 |
| Never Married | 0.98 (0.95, 1.02) | 0.02 | 0.333 | 1.31 (1.17, 1.47) | 0.08 | <0.001 |
|  |  |  |  |  |  |  |
| **Num. Elixhauser comorbidities in year prior** | 1.13 (1.12, 1.13) | <0.01 | <0.001 | 1.37 (1.33, 1.4) | 0.02 | <0.001 |
|  |  |  |  |  |  |  |
| **Mental health Ambulatory use in year prior (per 10 days)** | 0.87 (0.84, 0.9) | 0.01 | <0.001 | 1 (0.98, 1.02) | 0.01 | 0.639 |
|  |  |  |  |  |  |  |
| **Involuntary mental health admissions in year prior (per 10 admissions)** | 1 (0.99, 1) | <0.01 | 0.39 | 1 (0.99, 1.01) | 0.01 | 0.93 |
|  |  |  |  |  |  |  |
| **Emergency department presentations in year prior (per 10 presentations)^$^** | 1.6 (1.52, 1.69) | 0.04 | <0.001 | 1.23 (1.16, 1.31) | 0.04 | <0.001 |
|  |  |  |  |  |  |  |
| **History of depression** | 0.98 (0.96, 1) | 0.01 | 0.068 | 1.01 (0.93, 1.1) | 0.04 | 0.776 |
|  |  |  |  |  |  |  |
| **History of drug or alcohol abuse** | 1.02 (0.99, 1.04) | 0.01 | 0.202 | 1.43 (1.32, 1.55) | 0.06 | <0.001 |
|  |  |  |  |  |  |  |
| **History of psychotic disorder** | 0.83 (0.81, 0.86) | 0.01 | <0.001 | 1.13 (1.03, 1.25) | 0.05 | 0.009 |
|  |  |  |  |  |  |  |
| **History of anxiety disorder** | 0.9 (0.88, 0.92) | 0.01 | <0.001 | 0.95 (0.88, 1.03) | 0.04 | 0.202 |
|  |  |  |  |  |  |  |
| **History of delirium** | 1.33 (1.3, 1.35) | 0.01 | <0.001 | 1.6 (1.41, 1.81) | 0.1 | <0.001 |
|  |  |  |  |  |  |  |
| **History of behavioural problems** | 1.3 (1.27, 1.34) | 0.02 | <0.001 | 1.13 (1.04, 1.23) | 0.05 | 0.003 |
|  |  |  |  |  |  |  |
| **History of personality disorders** | 0.89 (0.83, 0.94) | 0.03 | <0.001 | 0.95 (0.85, 1.05) | 0.05 | 0.327 |
| ^✝^ “Unknown” category excluded from output | | | | | | |
| ^$^ To allow for model convergence, if someone had over 100 presentations to ED in a single year, we treated them as having at most 101 presentations in that year. | | | | | | |

## Figure S6: Comparison of age at first presentation for dementia cohort (left) and self-harm cohort (right) for whole cohort analysis (without mild cognitive impairment as an indicator of dementia)


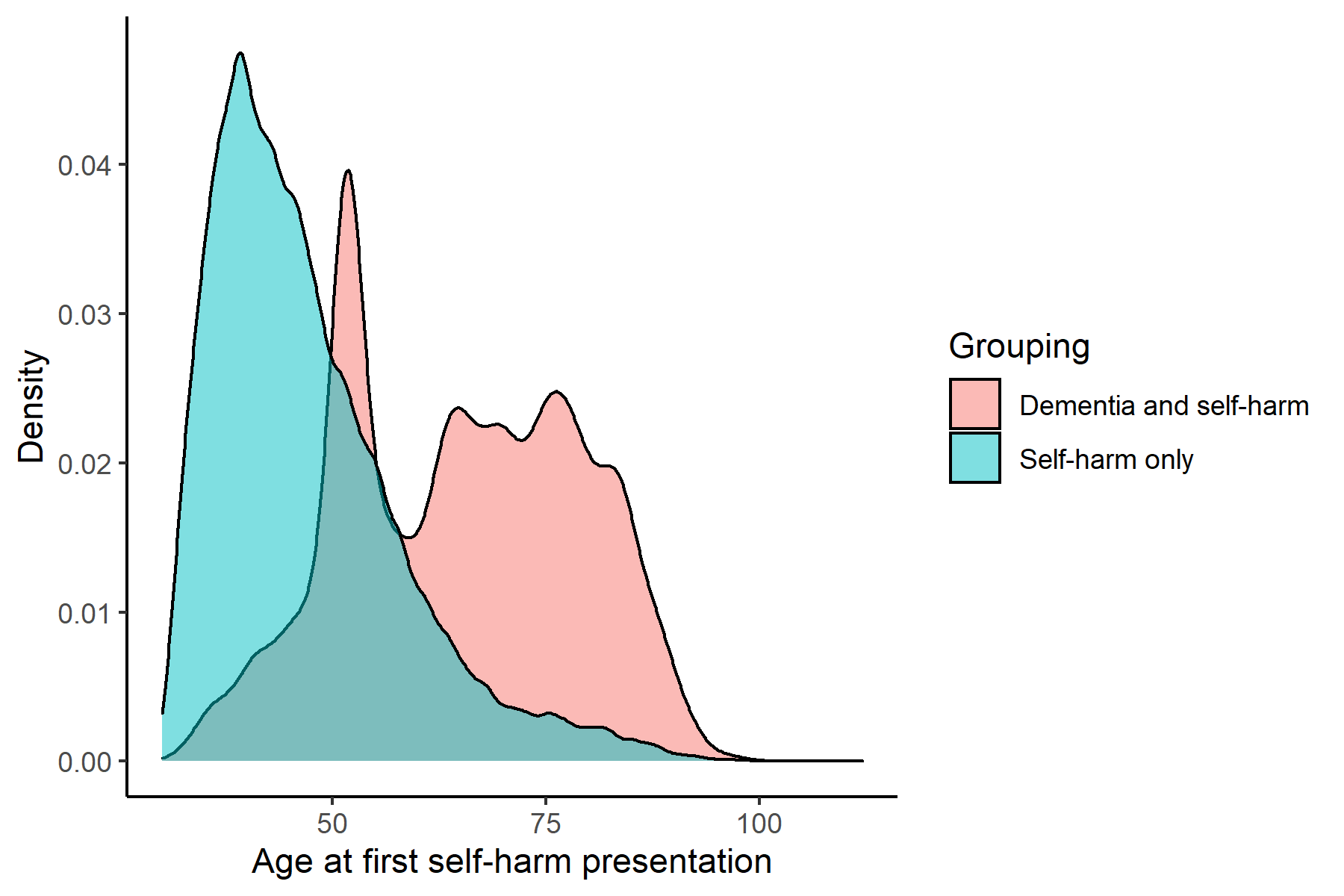

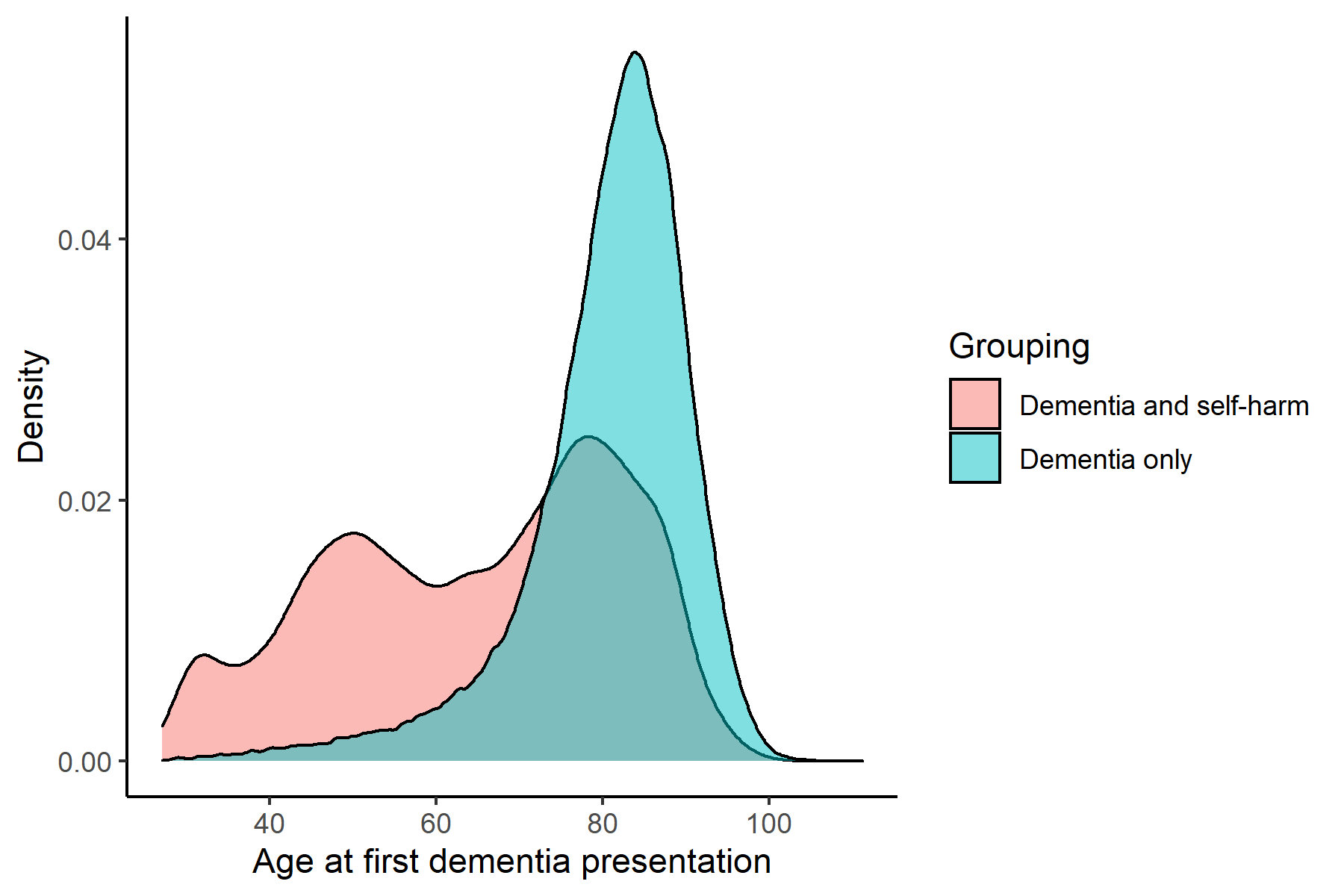


## Table S8: Predictors of repeat self-harm in the dementia and self-harm subgroup with age as the time scale – individually adjusted model

**(without mild cognitive impairment as an indicator of dementia)**

| **Variable** | **Incident Rate Ratio (95% CI)** | **Standard Error** | **p value** |
| --- | --- | --- | --- |
| **MH Ambulatory use in year prior (per 10 days)** | 0.99 (0.96, 1.02) | 0.02 | 0.56 |

Other model variables have been excluded due to a lack of interpretability

## Table S9: Predictors of repeat self-harm in the dementia and self-harm subgroup with age as the time scale – fully adjusted model

**(without mild cognitive impairment as an indicator of dementia)**

| **Variable** | **Incident Rate Ratio (95% CI)** | **Standard Error** | **p value** |
| --- | --- | --- | --- |
| **Sex (Female)**^✝^ | 0.54 (0.29, 1) | 0.17 | 0.051 |
|  |  |  |  |
| **Remoteness**^✝^ |  |  |  |
| Major cities (Reference) | 1 | … | … |
| Inner Regional | 2.21 (1.04, 4.68) | 0.85 | 0.039 |
| Outer regional and beyond | 5.21 (1.2, 22.6) | 3.9 | 0.027 |
|  |  |  |  |
| **Index of relative socioeconomic disadvantage quintile**^✝^ |  |  |  |
| 1-2 (Most disadvantaged) | 1 | … | … |
| 3-4 | 0.79 (0.38, 1.63) | 0.29 | 0.53 |
| 5-6 | 1.15 (0.31, 4.24) | 0.77 | 0.831 |
| 7-8 | 0.69 (0.22, 2.17) | 0.4 | 0.522 |
| 9-10 (Least disadvantaged) | 0.98 (0.37, 2.61) | 0.49 | 0.967 |
|  |  |  |  |
| **Marital Status**^✝^ |  |  |  |
| Married/De Facto | 1 | … | … |
| Divorced/Widowed/Separated | 0.41 (0.24, 0.69) | 0.11 | 0.001 |
| Never Married | 0.29 (0.12, 0.69) | 0.13 | 0.006 |
|  |  |  |  |
| **Num. Elixhauser comorbidities in year prior** | 1.25 (1.1, 1.41) | 0.08 | 0.001 |
|  |  |  |  |
| **Mental health Ambulatory use in year prior (per 10 days)** | 0.98 (0.95, 1.01) | 0.01 | 0.246 |
|  |  |  |  |
| **Involuntary mental health admissions in year prior (per 10 admissions)** | 0.8 (0.65, 1) | 0.09 | 0.048 |
|  |  |  |  |
| **Emergency department presentations in year prior  (per 10 presentations)^$^** | 1.34 (1.14, 1.58) | 0.11 | <0.001 |
|  |  |  |  |
| **History of depression** | 0.47 (0.27, 0.83) | 0.14 | 0.009 |
|  |  |  |  |
| **History of drug or alcohol abuse** | 0.7 (0.4, 1.25) | 0.2 | 0.228 |
|  |  |  |  |
| **History of psychotic disorder** | 1.53 (0.93, 2.51) | 0.39 | 0.094 |
|  |  |  |  |
| **History of anxiety disorder** | 1.12 (0.65, 1.93) | 0.31 | 0.693 |
|  |  |  |  |
| **History of delirium** | 1.14 (0.67, 1.95) | 0.31 | 0.619 |
|  |  |  |  |
| **History of behavioural problems** | 1.45 (0.95, 2.23) | 0.32 | 0.088 |
|  |  |  |  |
| **History of personality disorders** | 1.48 (0.75, 2.95) | 0.52 | 0.261 |
| ^✝^ “Unknown” category excluded from output | | | |
| ^$^ To allow for model convergence, if someone had over 100 presentations to ED in a single year, we treated them as having at most 101 presentations in that year. | | | |

# Full regression results of comparison of rate of death between cohorts

## Table S10: Survival analysis results for comparison of rate of death between cohorts (1-nearest matching)

|  | **Dementia cohort** | | | **Self-harm cohort** | | |
| --- | --- | --- | --- | --- | --- | --- |
| **Variable** | **Incident Rate Ratio (95% CI)** | **Standard Error** | **p value** | **Incident Rate Ratio (95% CI)** | **Standard Error** | **p value** |
| **Cohort/subgroup** |  |  |  |  |  |  |
| Dementia and Self-harm | 1 | … | … | 1 | … | … |
| Dementia only | 0.8 (0.67, 0.94) | 0.07 | 0.007 | … | … | … |
| Self-harm only | … | … | … | 0.4 (0.33, 0.49) | 0.04 | <0.001 |
|  |  |  |  |  |  |  |
| **Sex (Female)**^✝^ | 1 (1, 1) | <0.01 | <0.001 | 1 (1, 1) | <0.01 | 0.001 |
|  |  |  |  |  |  |  |
| **Remoteness**^✝^ |  |  |  |  |  |  |
| Major cities (Reference) | 1 | … | … | 1 | … | … |
| Inner Regional | 1.21 (0.99, 1.47) | 0.12 | 0.057 | 1.08 (0.86, 1.37) | 0.13 | 0.5 |
| Outer regional and beyond | 1.39 (0.97, 2) | 0.26 | 0.076 | 1.44 (1.01, 2.05) | 0.26 | 0.041 |
|  |  |  |  |  |  |  |
| **Index of relative socioeconomic disadvantage quintile**^✝^ |  |  |  |  |  |  |
| 1-2 (Most disadvantaged) | 1 | … | … | 1 | … | … |
| 3-4 | 0.87 (0.7, 1.06) | 0.09 | 0.172 | 1 (0.79, 1.28) | 0.12 | 0.991 |
| 5-6 | 0.96 (0.76, 1.21) | 0.12 | 0.718 | 0.88 (0.66, 1.16) | 0.12 | 0.349 |
| 7-8 | 0.93 (0.72, 1.21) | 0.12 | 0.61 | 0.94 (0.7, 1.27) | 0.15 | 0.691 |
| 9-10 (Least disadvantaged) | 0.97 (0.76, 1.22) | 0.12 | 0.774 | 0.98 (0.74, 1.3) | 0.14 | 0.891 |
|  |  |  |  |  |  |  |
| **Marital Status**^✝^ |  |  |  |  |  |  |
| Married/De Facto | 1 | … | … | 1 | … | … |
| Divorced/Widowed/Separated | 0.88 (0.75, 1.04) | 0.07 | 0.143 | 0.89 (0.74, 1.07) | 0.08 | 0.221 |
| Never Married | 1.15 (0.89, 1.5) | 0.15 | 0.29 | 1.27 (0.93, 1.74) | 0.2 | 0.131 |
|  |  |  |  |  |  |  |
| **Num. Elixhauser comorbidities in year prior** | 1.18 (1.13, 1.23) | 0.03 | <0.001 | 1.21 (1.16, 1.26) | 0.03 | <0.001 |
|  |  |  |  |  |  |  |
| **Mental health Ambulatory use in year prior (per 10 days)** | 0.88 (0.77, 1) | 0.06 | 0.054 | 0.85 (0.76, 0.96) | 0.05 | 0.008 |
|  |  |  |  |  |  |  |
| **Involuntary mental health admissions in year prior (per 10 admissions)** | 0.98 (0.93, 1.03) | 0.03 | 0.458 | 1.03 (0.98, 1.09) | 0.03 | 0.281 |
|  |  |  |  |  |  |  |
| **Emergency department presentations in year prior (per 10 presentations)^$^** | 1.29 (1.1, 1.51) | 0.1 | 0.002 | 1.18 (1.01, 1.37) | 0.09 | 0.032 |
|  |  |  |  |  |  |  |
| **History of depression** | 0.84 (0.7, 1) | 0.07 | 0.047 | 0.91 (0.75, 1.1) | 0.09 | 0.333 |
|  |  |  |  |  |  |  |
| **History of drug or alcohol abuse** | 1.02 (0.84, 1.25) | 0.1 | 0.811 | 1.4 (1.13, 1.73) | 0.15 | 0.002 |
|  |  |  |  |  |  |  |
| **History of psychotic disorder** | 0.9 (0.74, 1.09) | 0.09 | 0.292 | 0.9 (0.72, 1.12) | 0.1 | 0.329 |
|  |  |  |  |  |  |  |
| **History of anxiety disorder** | 0.77 (0.64, 0.94) | 0.08 | 0.01 | 1.04 (0.87, 1.25) | 0.1 | 0.661 |
|  |  |  |  |  |  |  |
| **History of delirium** | 1.39 (1.16, 1.68) | 0.13 | <0.001 | 1.46 (1.19, 1.78) | 0.15 | <0.001 |
|  |  |  |  |  |  |  |
| **History of behavioural problems** | 1.06 (0.86, 1.31) | 0.11 | 0.573 | 1.07 (0.87, 1.31) | 0.11 | 0.509 |
|  |  |  |  |  |  |  |
| **History of personality disorders** | 0.83 (0.6, 1.14) | 0.14 | 0.257 | 0.62 (0.45, 0.86) | 0.1 | 0.004 |
| ^✝^ “Unknown” category excluded from output | | | | | | |
| ^$^ To allow for model convergence, if someone had over 100 presentations to ED in a single year, we treated them as having at most 101 presentations in that year. | | | | | | |

## Figure S7: Comparison of age at first presentation for dementia (left) and self-harm (right) for 1-nearest matching analysis


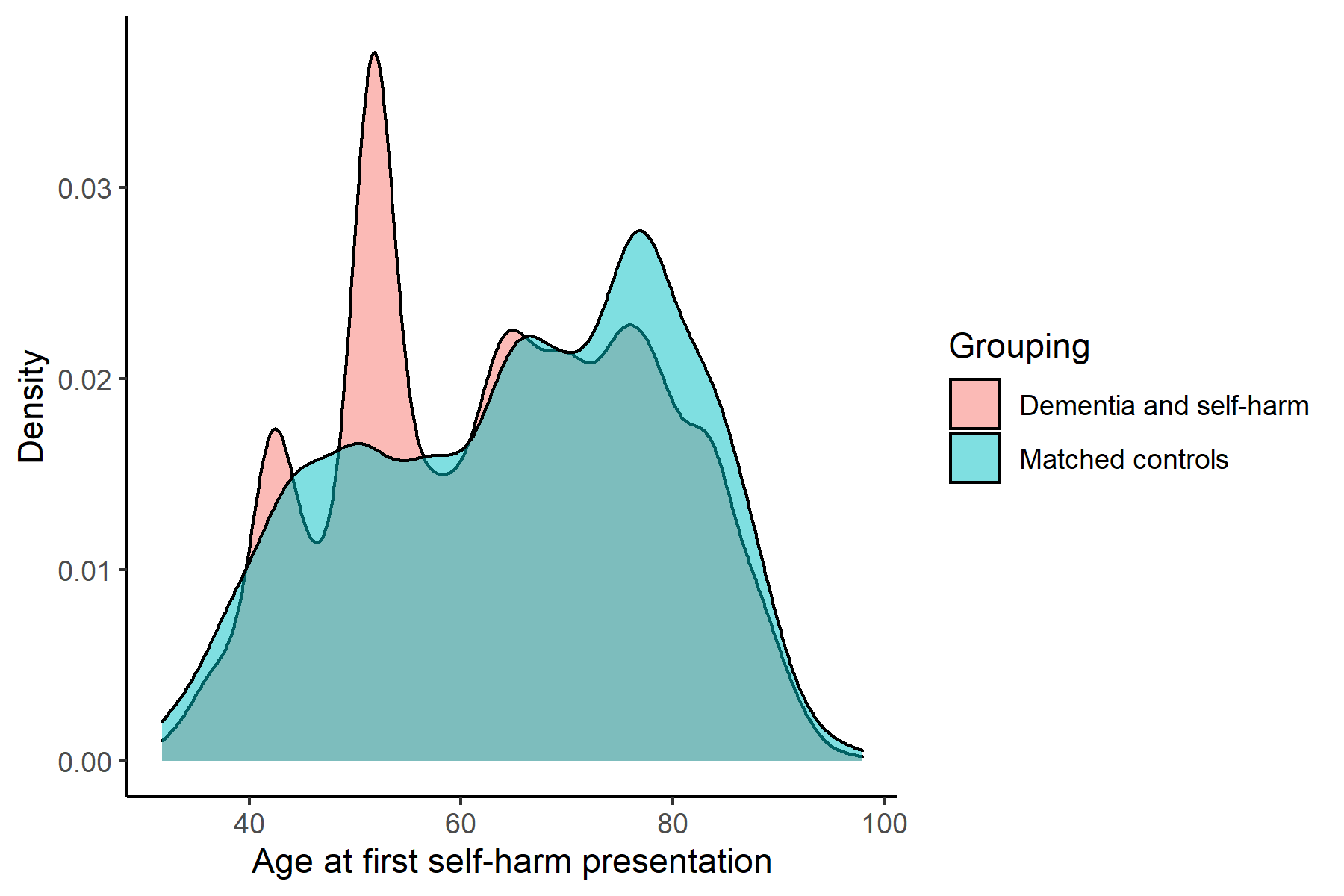


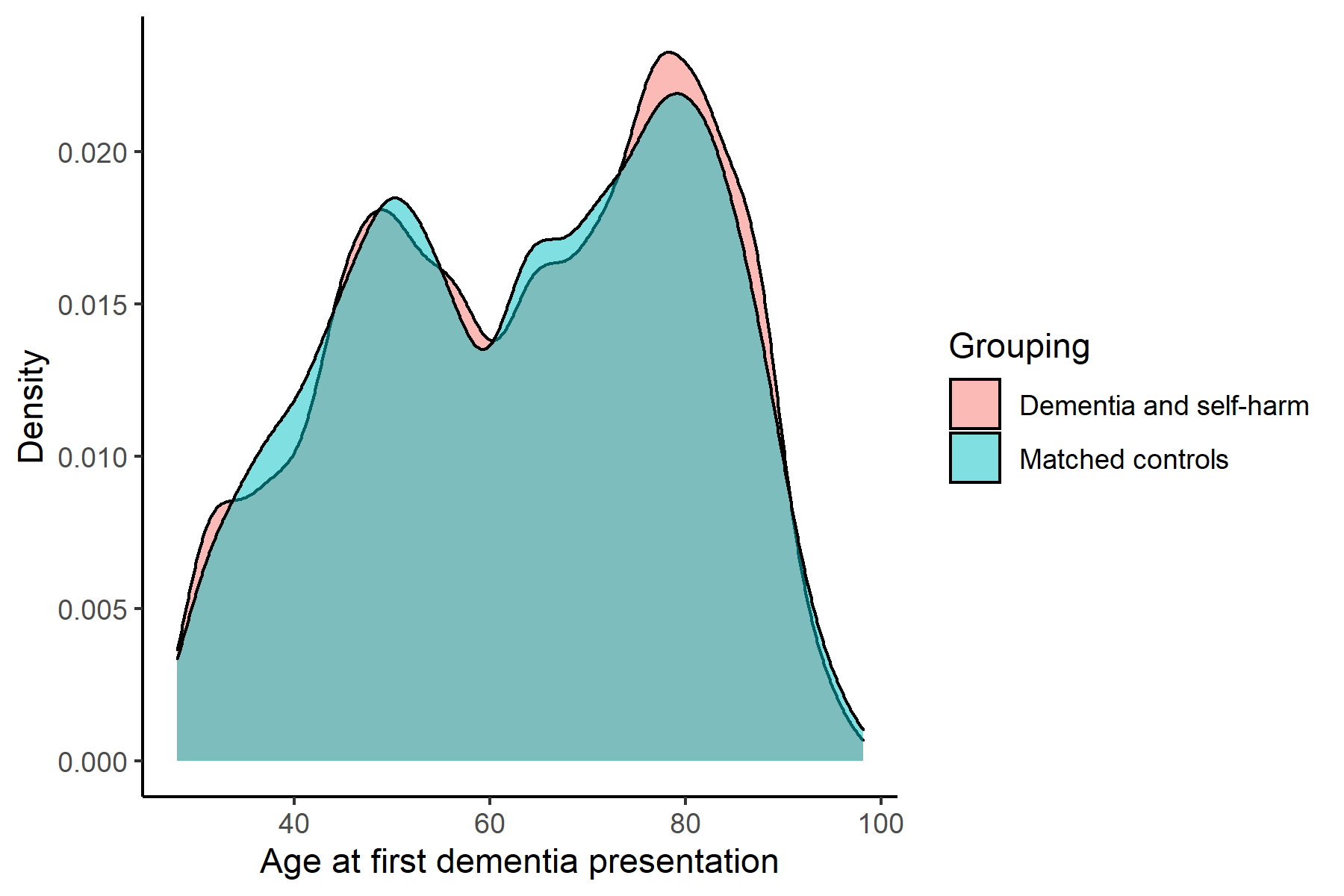


## Table S11: Survival analysis results for comparison of rate of death between cohorts (5-nearest matching)

|  | **Dementia cohort** | | | **Self-harm cohort** | | |
| --- | --- | --- | --- | --- | --- | --- |
| **Variable** | **Incident Rate Ratio (95% CI)** | **Standard Error** | **p value** | **Incident Rate Ratio (95% CI)** | **Standard Error** | **p value** |
| **Cohort/subgroup** |  |  |  |  |  |  |
| Dementia and Self-harm | 1 | … | … | 1 | … | … |
| Dementia only | 1.08 (0.94, 1.23) | 0.07 | 0.269 | … | … | … |
| Self-harm only | … | … | … | 0.61 (0.53, 0.71) | 0.05 | <0.001 |
|  |  |  |  |  |  |  |
| **Sex (Female)**^✝^ | 1 (1, 1) | <0.01 | <0.001 | 1 (1, 1) | <0.01 | <0.001 |
|  |  |  |  |  |  |  |
| **Remoteness**^✝^ |  |  |  |  |  |  |
| Major cities (Reference) | 1 | … | … | 1 | … | … |
| Inner Regional | 1.1 (0.98, 1.23) | 0.06 | 0.095 | 1.04 (0.9, 1.2) | 0.08 | 0.593 |
| Outer regional and beyond | 1.19 (0.99, 1.42) | 0.11 | 0.058 | 1.12 (0.9, 1.41) | 0.13 | 0.317 |
|  |  |  |  |  |  |  |
| **Index of relative socioeconomic disadvantage quintile**^✝^ |  |  |  |  |  |  |
| 1-2 (Most disadvantaged) | 1 | … | … | 1 | … | … |
| 3-4 | 0.85 (0.76, 0.95) | 0.05 | 0.006 | 1 (0.86, 1.17) | 0.08 | 0.996 |
| 5-6 | 0.93 (0.82, 1.06) | 0.06 | 0.292 | 0.95 (0.8, 1.14) | 0.09 | 0.592 |
| 7-8 | 0.88 (0.76, 1.02) | 0.07 | 0.103 | 0.86 (0.71, 1.06) | 0.09 | 0.159 |
| 9-10 (Least disadvantaged) | 1 (0.87, 1.14) | 0.07 | 0.949 | 0.89 (0.74, 1.09) | 0.09 | 0.258 |
|  |  |  |  |  |  |  |
| **Marital Status**^✝^ |  |  |  |  |  |  |
| Married/De Facto | 1 | … | … | 1 | … | … |
| Divorced/Widowed/Separated | 0.98 (0.89, 1.07) | 0.05 | 0.615 | 1.01 (0.9, 1.14) | 0.06 | 0.873 |
| Never Married | 1.19 (1.03, 1.37) | 0.09 | 0.02 | 1.14 (0.92, 1.42) | 0.13 | 0.228 |
|  |  |  |  |  |  |  |
| **Num. Elixhauser comorbidities in year prior** | 1.17 (1.14, 1.2) | 0.01 | <0.001 | 1.29 (1.26, 1.33) | 0.02 | <0.001 |
|  |  |  |  |  |  |  |
| **Mental health Ambulatory use in year prior (per 10 days)** | 0.83 (0.74, 0.94) | 0.05 | 0.004 | 0.91 (0.85, 0.98) | 0.03 | 0.008 |
|  |  |  |  |  |  |  |
| **Involuntary mental health admissions in year prior (per 10 admissions)** | 0.98 (0.95, 1.02) | 0.02 | 0.354 | 1.02 (0.97, 1.08) | 0.03 | 0.46 |
|  |  |  |  |  |  |  |
| **Emergency department presentations in year prior (per 10 presentations)^$^** | 1.33 (1.16, 1.53) | 0.1 | <0.001 | 1.26 (1.13, 1.4) | 0.07 | <0.001 |
|  |  |  |  |  |  |  |
| **History of depression** | 0.9 (0.8, 1) | 0.05 | 0.056 | 0.99 (0.88, 1.12) | 0.06 | 0.906 |
|  |  |  |  |  |  |  |
| **History of drug or alcohol abuse** | 1.07 (0.95, 1.2) | 0.06 | 0.273 | 1.16 (1.02, 1.32) | 0.08 | 0.023 |
|  |  |  |  |  |  |  |
| **History of psychotic disorder** | 0.81 (0.72, 0.92) | 0.05 | 0.001 | 0.92 (0.78, 1.07) | 0.07 | 0.275 |
|  |  |  |  |  |  |  |
| **History of anxiety disorder** | 0.78 (0.69, 0.89) | 0.05 | <0.001 | 0.98 (0.87, 1.1) | 0.06 | 0.681 |
|  |  |  |  |  |  |  |
| **History of delirium** | 1.35 (1.21, 1.5) | 0.07 | <0.001 | 1.53 (1.32, 1.78) | 0.12 | <0.001 |
|  |  |  |  |  |  |  |
| **History of behavioural problems** | 1.17 (1.03, 1.33) | 0.08 | 0.015 | 1.07 (0.94, 1.21) | 0.07 | 0.323 |
|  |  |  |  |  |  |  |
| **History of personality disorders** | 0.84 (0.67, 1.05) | 0.09 | 0.122 | 0.73 (0.58, 0.91) | 0.08 | 0.005 |
| ^✝^ “Unknown” category excluded from output | | | | | | |
| ^$^ To allow for model convergence, if someone had over 100 presentations to ED in a single year, we treated them as having at most 101 presentations in that year. | | | | | | |

## Figure S8: Comparison of age at first presentation for dementia (left) and self-harm (right) for 5-nearest matching analysis


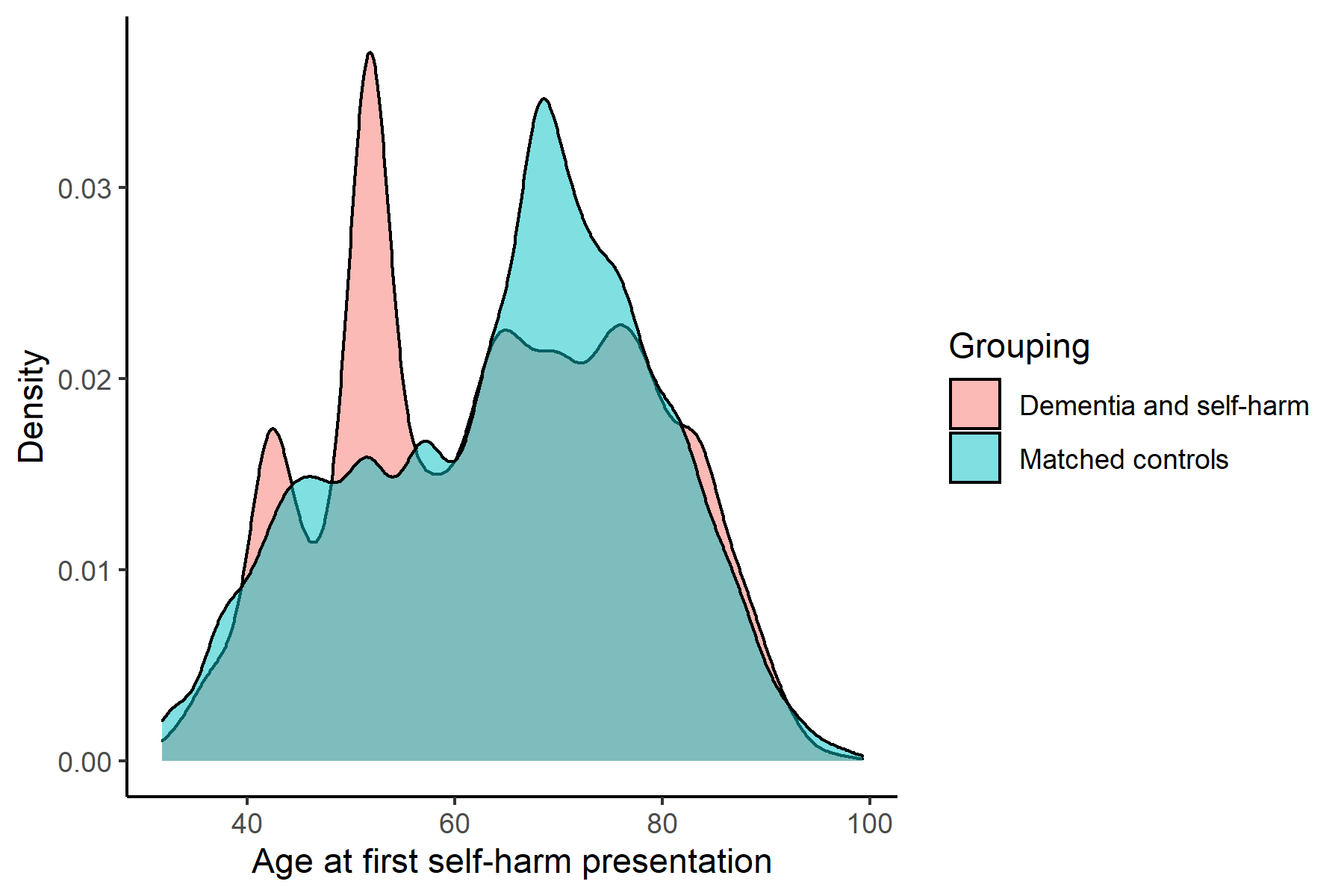

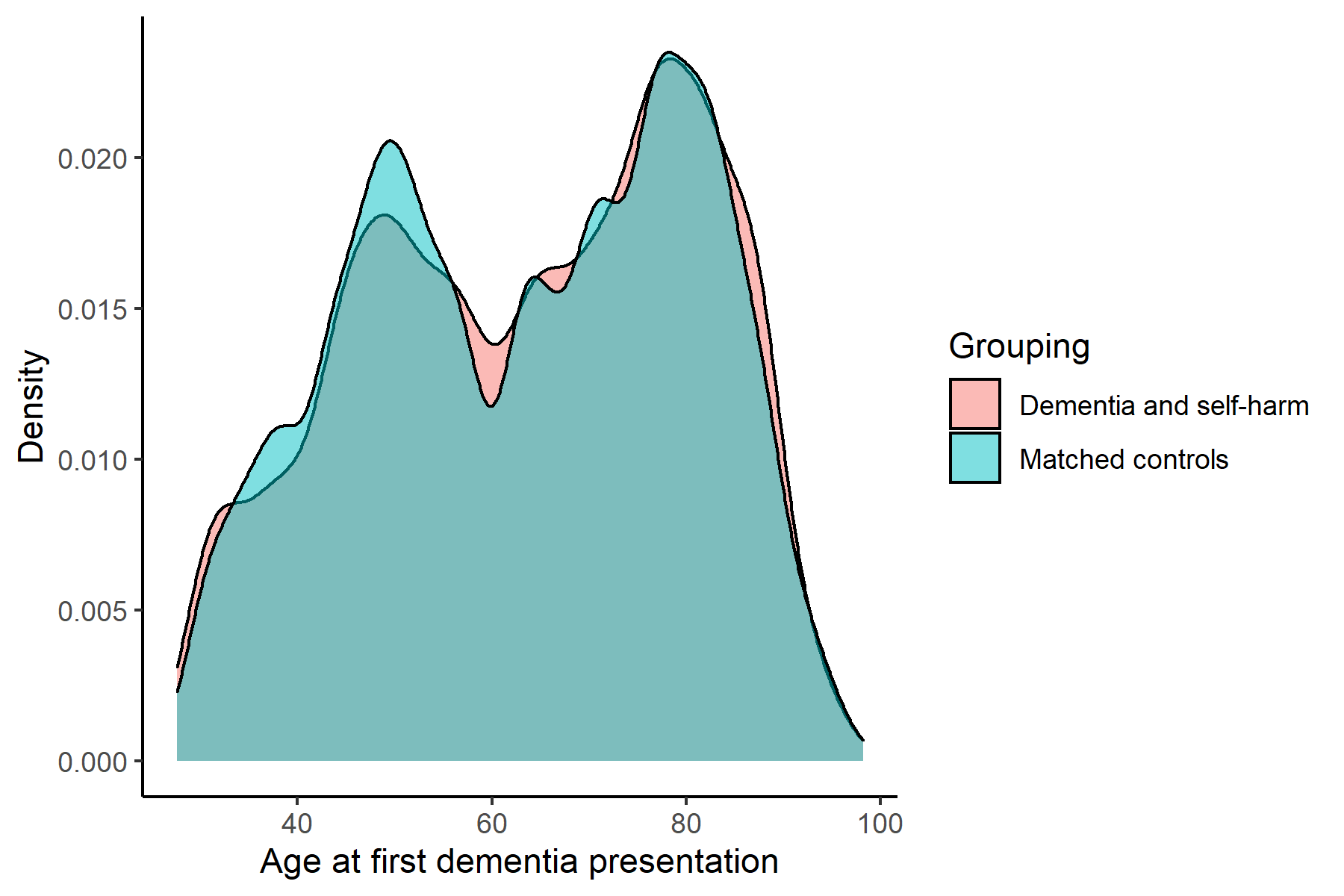


## Table S12: Survival analysis results for comparison of rate of death between cohorts (20-nearest matching)

|  | **Dementia cohort** | | | **Self-harm cohort** | | |
| --- | --- | --- | --- | --- | --- | --- |
| **Variable** | **Incident Rate Ratio (95% CI)** | **Standard Error** | **p value** | **Incident Rate Ratio (95% CI)** | **Standard Error** | **p value** |
| **Cohort/subgroup** |  |  |  |  |  |  |
| Dementia and Self-harm | 1 | … | … | 1 | … | … |
| Dementia only | 1.3 (1.15, 1.47) | 0.08 | <0.001 | … | … | … |
| Self-harm only | … | … | … | 0.69 (0.6, 0.81) | 0.05 | <0.001 |
|  |  |  |  |  |  |  |
| **Sex (Female)**^✝^ | 0.82 (0.78, 0.86) | 0.02 | <0.001 | 1 (1, 1) | <0.01 | 0.029 |
|  |  |  |  |  |  |  |
| **Remoteness**^✝^ |  |  |  |  |  |  |
| Major cities (Reference) | 1 | … | … | 1 | … | … |
| Inner Regional | 1.11 (1.04, 1.18) | 0.03 | 0.001 | 1.08 (0.98, 1.2) | 0.06 | 0.133 |
| Outer regional and beyond | 1.08 (0.98, 1.18) | 0.05 | 0.122 | 1.07 (0.9, 1.25) | 0.09 | 0.448 |
|  |  |  |  |  |  |  |
| **Index of relative socioeconomic disadvantage quintile**^✝^ |  |  |  |  |  |  |
| 1-2 (Most disadvantaged) | 1 | … | … | 1 | … | … |
| 3-4 | 0.94 (0.89, 1) | 0.03 | 0.065 | 1.03 (0.92, 1.15) | 0.06 | 0.584 |
| 5-6 | 0.96 (0.9, 1.03) | 0.03 | 0.285 | 1.02 (0.89, 1.16) | 0.07 | 0.784 |
| 7-8 | 0.92 (0.85, 1) | 0.04 | 0.044 | 0.9 (0.77, 1.05) | 0.07 | 0.19 |
| 9-10 (Least disadvantaged) | 0.95 (0.88, 1.02) | 0.04 | 0.175 | 0.99 (0.85, 1.14) | 0.07 | 0.866 |
|  |  |  |  |  |  |  |
| **Marital Status**^✝^ |  |  |  |  |  |  |
| Married/De Facto | 1 | … | … | 1 | … | … |
| Divorced/Widowed/Separated | 1.01 (0.96, 1.06) | 0.03 | 0.802 | 1.13 (1.04, 1.24) | 0.05 | 0.006 |
| Never Married | 1.12 (1.04, 1.22) | 0.05 | 0.004 | 1.33 (1.17, 1.52) | 0.09 | <0.001 |
|  |  |  |  |  |  |  |
| **Num. Elixhauser comorbidities in year prior** | 1.17 (1.16, 1.19) | 0.01 | <0.001 | 1.34 (1.31, 1.38) | 0.02 | <0.001 |
|  |  |  |  |  |  |  |
| **Mental health Ambulatory use in year prior (per 10 days)** | 0.89 (0.83, 0.96) | 0.03 | 0.001 | 0.98 (0.95, 1.01) | 0.02 | 0.191 |
|  |  |  |  |  |  |  |
| **Involuntary mental health admissions in year prior (per 10 admissions)** | 0.99 (0.98, 1) | <0.01 | 0.142 | 1 (0.99, 1.01) | 0.01 | 0.978 |
|  |  |  |  |  |  |  |
| **Emergency department presentations in year prior (per 10 presentations)^$^** | 1.4 (1.28, 1.53) | 0.06 | <0.001 | 1.26 (1.18, 1.33) | 0.04 | <0.001 |
|  |  |  |  |  |  |  |
| **History of depression** | 0.93 (0.88, 0.99) | 0.03 | 0.021 | 1.02 (0.93, 1.11) | 0.05 | 0.659 |
|  |  |  |  |  |  |  |
| **History of drug or alcohol abuse** | 1.11 (1.05, 1.19) | 0.04 | 0.001 | 1.4 (1.29, 1.53) | 0.06 | <0.001 |
|  |  |  |  |  |  |  |
| **History of psychotic disorder** | 0.85 (0.79, 0.91) | 0.03 | <0.001 | 1.11 (0.99, 1.24) | 0.06 | 0.067 |
|  |  |  |  |  |  |  |
| **History of anxiety disorder** | 0.83 (0.77, 0.89) | 0.03 | <0.001 | 0.93 (0.85, 1.01) | 0.04 | 0.095 |
|  |  |  |  |  |  |  |
| **History of delirium** | 1.35 (1.27, 1.43) | 0.04 | <0.001 | 1.6 (1.4, 1.82) | 0.11 | <0.001 |
|  |  |  |  |  |  |  |
| **History of behavioural problems** | 1.24 (1.15, 1.33) | 0.05 | <0.001 | 1.11 (1.01, 1.21) | 0.05 | 0.035 |
|  |  |  |  |  |  |  |
| **History of personality disorders** | 0.93 (0.81, 1.06) | 0.06 | 0.276 | 0.86 (0.75, 0.98) | 0.06 | 0.023 |
| ^✝^ “Unknown” category excluded from output | | | | | | |
| ^$^ To allow for model convergence, if someone had over 100 presentations to ED in a single year, we treated them as having at most 101 presentations in that year. | | | | | | |

## Figure S9: Comparison of age at first presentation for dementia (left) and self-harm (right) for 20-nearest matching analysis


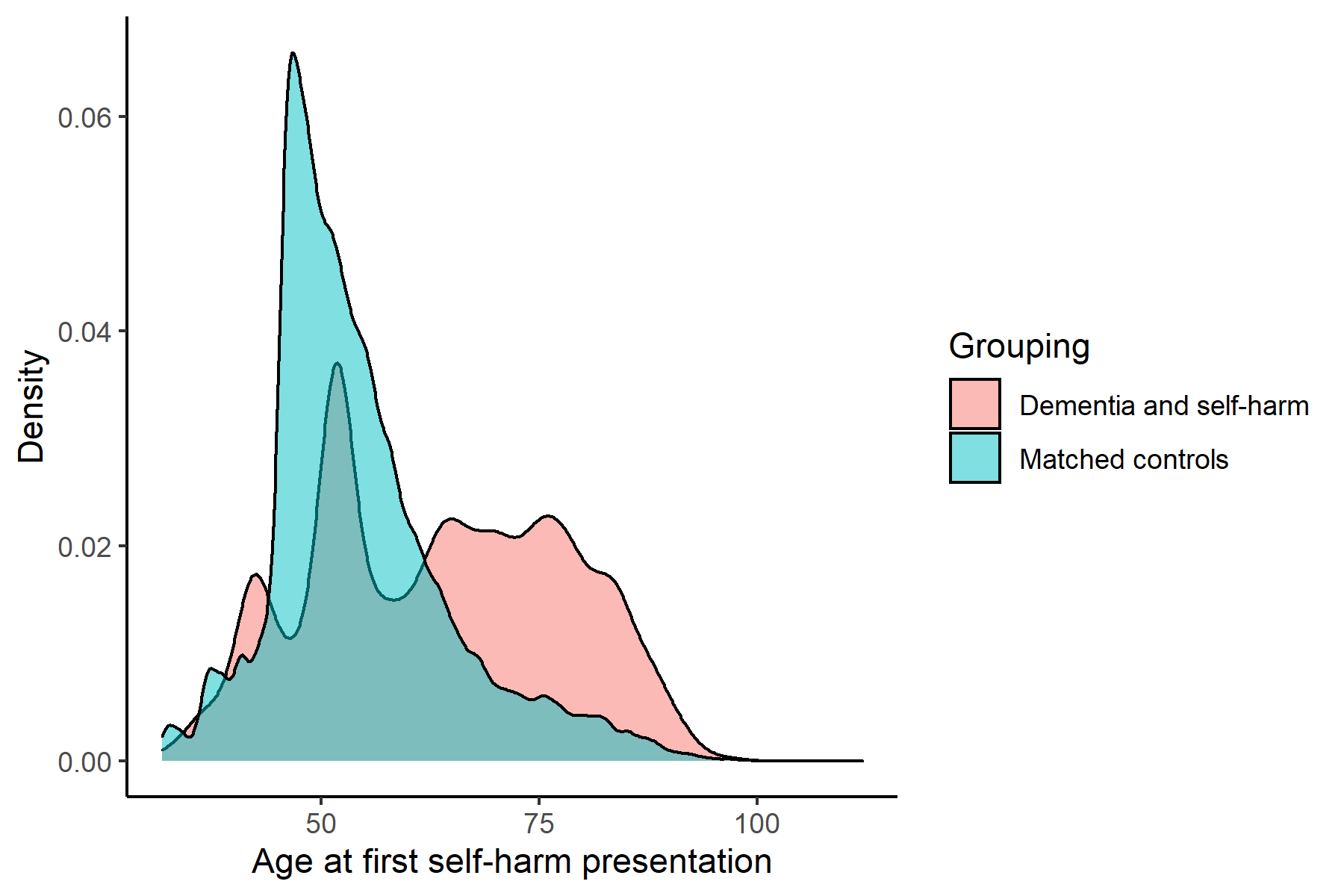

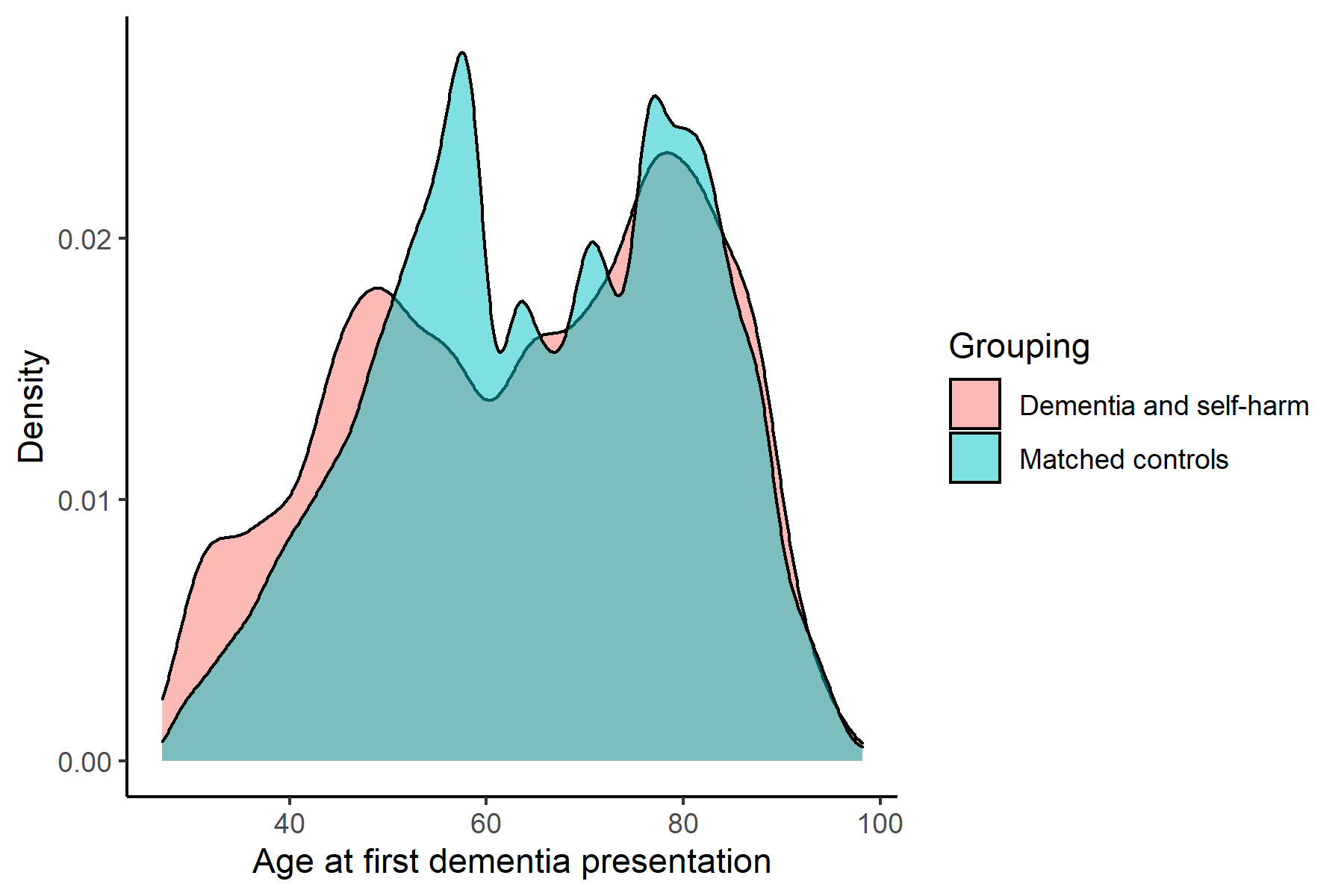


## Table S13: Survival analysis results for comparison of rate of death between cohorts (whole cohort)

|  | **Dementia cohort** | | | **Self-harm cohort** | | |
| --- | --- | --- | --- | --- | --- | --- |
| **Variable** | **Incident Rate Ratio (95% CI)** | **Standard Error** | **p value** | **Incident Rate Ratio (95% CI)** | **Standard Error** | **p value** |
| **Cohort/subgroup** |  |  |  |  |  |  |
| Dementia and Self-harm | 1 | … | … | 1 | … | … |
| Dementia only | 1.61 (1.31, 1.99) | 0.17 | <0.001 | … | … | … |
| Self-harm only | … | … | … | 0.73 (0.62, 0.85) | 0.06 | <0.001 |
|  |  |  |  |  |  |  |
| **Sex (Female)**^✝^ | 0.75 (0.74, 0.76) | 0.01 | <0.001 | 1 (1, 1) | <0.01 | 0.975 |
|  |  |  |  |  |  |  |
| **Remoteness**^✝^ |  |  |  |  |  |  |
| Major cities (Reference) | 1 | … | … | 1 | … | … |
| Inner Regional | 1.1 (1.08, 1.12) | 0.01 | <0.001 | 1.06 (0.97, 1.17) | 0.05 | 0.188 |
| Outer regional and beyond | 1.09 (1.06, 1.12) | 0.02 | <0.001 | 1.02 (0.88, 1.19) | 0.08 | 0.762 |
|  |  |  |  |  |  |  |
| **Index of relative socioeconomic disadvantage quintile**^✝^ |  |  |  |  |  |  |
| 1-2 (Most disadvantaged) | 1 | … | … | 1 | … | … |
| 3-4 | 0.98 (0.97, 1) | 0.01 | 0.092 | 1 (0.9, 1.1) | 0.05 | 0.96 |
| 5-6 | 0.96 (0.94, 0.98) | 0.01 | <0.001 | 0.98 (0.87, 1.1) | 0.06 | 0.708 |
| 7-8 | 0.96 (0.94, 0.99) | 0.01 | 0.002 | 0.87 (0.76, 1.01) | 0.06 | 0.063 |
| 9-10 (Least disadvantaged) | 0.91 (0.89, 0.93) | 0.01 | <0.001 | 0.95 (0.83, 1.09) | 0.07 | 0.453 |
|  |  |  |  |  |  |  |
| **Marital Status**^✝^ |  |  |  |  |  |  |
| Married/De Facto | 1 | … | … | 1 | … | … |
| Divorced/Widowed/Separated | 1.02 (1, 1.03) | 0.01 | 0.062 | 1.16 (1.06, 1.26) | 0.05 | 0.001 |
| Never Married | 0.98 (0.95, 1.01) | 0.02 | 0.215 | 1.31 (1.17, 1.47) | 0.08 | <0.001 |
|  |  |  |  |  |  |  |
| **Num. Elixhauser comorbidities in year prior** | 1.13 (1.12, 1.13) | <0.01 | <0.001 | 1.36 (1.33, 1.4) | 0.02 | <0.001 |
|  |  |  |  |  |  |  |
| **Mental health Ambulatory use in year prior (per 10 days)** | 0.83 (0.8, 0.87) | 0.02 | <0.001 | 1 (0.98, 1.02) | 0.01 | 0.808 |
|  |  |  |  |  |  |  |
| **Involuntary mental health admissions in year prior (per 10 admissions)** | 1 (1, 1) | <0.01 | 0.99 | 1 (0.98, 1.01) | 0.01 | 0.929 |
|  |  |  |  |  |  |  |
| **Emergency department presentations in year prior (per 10 presentations)^$^** | 1.61 (1.53, 1.69) | 0.04 | <0.001 | 1.24 (1.17, 1.31) | 0.04 | <0.001 |
|  |  |  |  |  |  |  |
| **History of depression** | 0.98 (0.96, 1) | 0.01 | 0.073 | 1.01 (0.93, 1.09) | 0.04 | 0.799 |
|  |  |  |  |  |  |  |
| **History of drug or alcohol abuse** | 1.02 (1, 1.05) | 0.01 | 0.114 | 1.43 (1.32, 1.55) | 0.06 | <0.001 |
|  |  |  |  |  |  |  |
| **History of psychotic disorder** | 0.84 (0.81, 0.86) | 0.01 | <0.001 | 1.14 (1.04, 1.26) | 0.06 | 0.008 |
|  |  |  |  |  |  |  |
| **History of anxiety disorder** | 0.9 (0.88, 0.92) | 0.01 | <0.001 | 0.95 (0.88, 1.03) | 0.04 | 0.185 |
|  |  |  |  |  |  |  |
| **History of delirium** | 1.34 (1.31, 1.36) | 0.01 | <0.001 | 1.61 (1.42, 1.82) | 0.1 | <0.001 |
|  |  |  |  |  |  |  |
| **History of behavioural problems** | 1.29 (1.26, 1.33) | 0.02 | <0.001 | 1.14 (1.05, 1.24) | 0.05 | 0.002 |
|  |  |  |  |  |  |  |
| **History of personality disorders** | 0.89 (0.84, 0.94) | 0.03 | <0.001 | 0.93 (0.84, 1.04) | 0.05 | 0.235 |
| ^✝^ “Unknown” category excluded from output | | | | | | |
| ^$^ To allow for model convergence, if someone had over 100 presentations to ED in a single year, we treated them as having at most 101 presentations in that year. | | | | | | |

## Figure S10: Comparison of age at first presentation for dementia cohort (left) and self-harm cohort (right) for whole cohort analysis


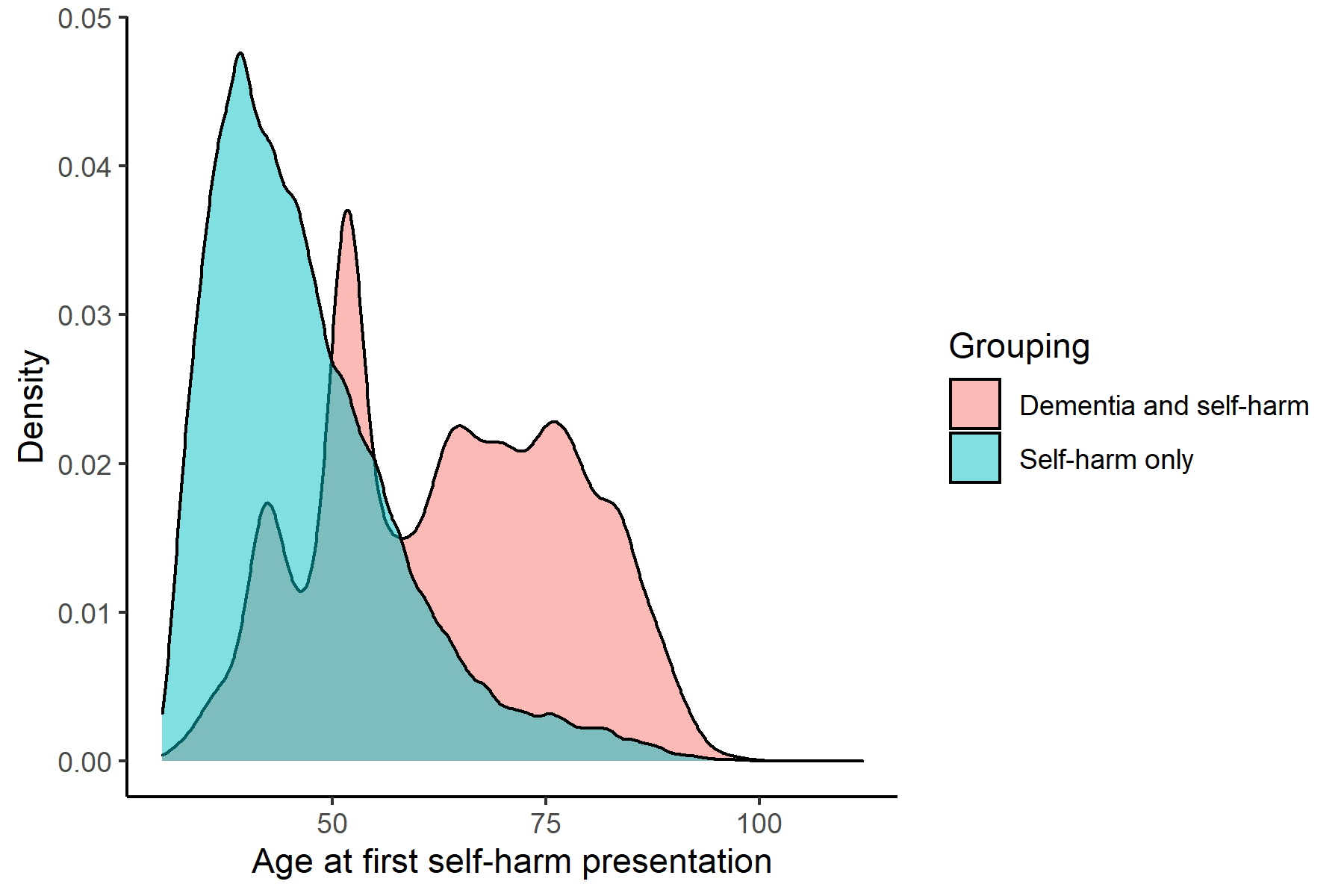

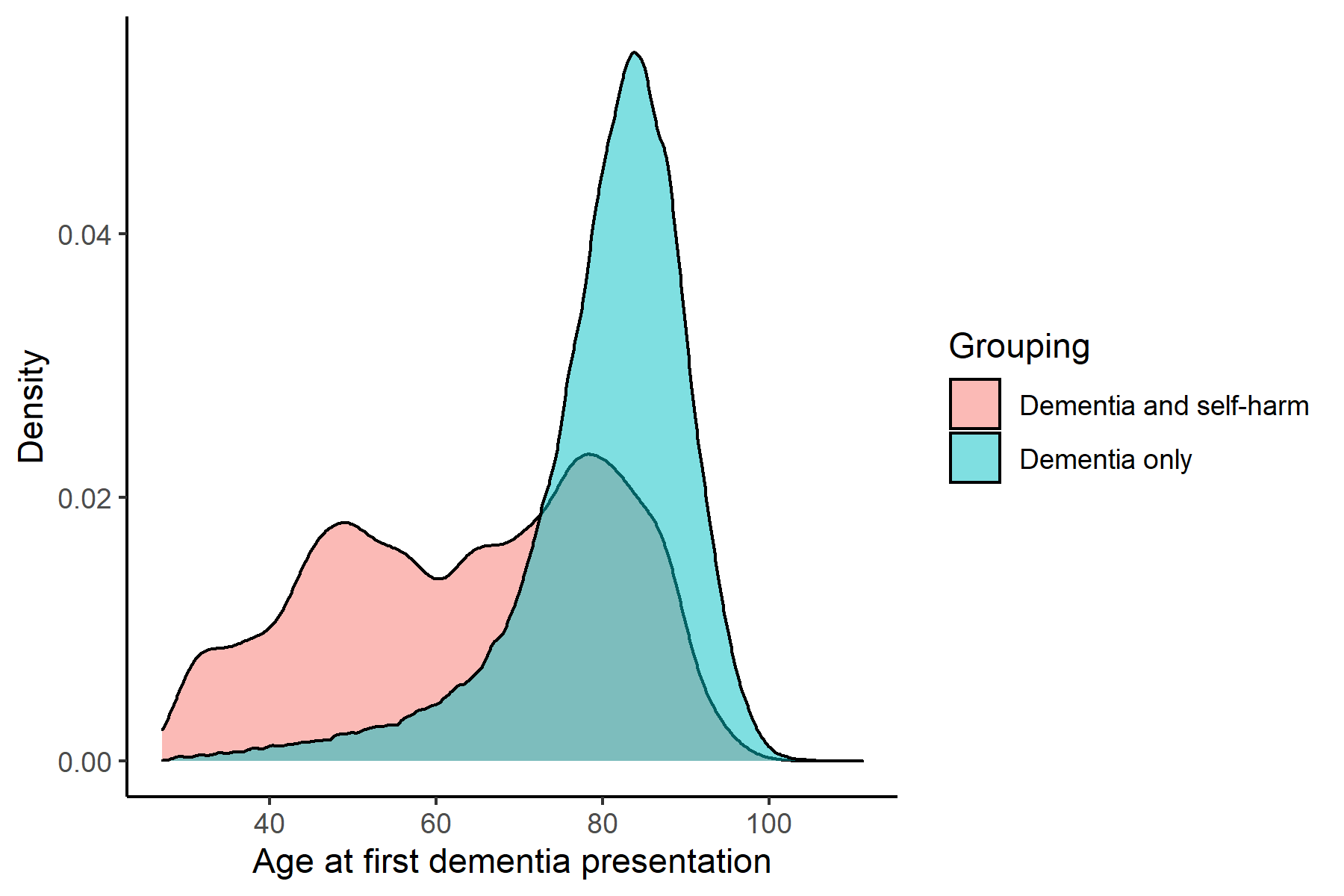


# Model results for repeat self-harm

## Table S14: Predictors of repeat self-harm in the dementia and self-harm subgroup with age as the time scale – individually adjusted model

| **Variable** | **Incident Rate Ratio (95% CI)** | **Standard Error** | **p value** |
| --- | --- | --- | --- |
| **MH Ambulatory use in year prior (per 10 days)** | 0.93 (0.88, 0.98) | 0.03 | 0.01 |

## Table S15: Predictors of repeat self-harm in the dementia and self-harm subgroup with age as the time scale – fully adjusted model

| **Variable** | **Incident Rate Ratio (95% CI)** | **Standard Error** | **p value** |
| --- | --- | --- | --- |
| **Sex (Female)**^✝^ | 0.37 (0.19, 0.72) | 0.13 | 0.003 |
|  |  |  |  |
| **Remoteness**^✝^ |  |  |  |
| Major cities (Reference) | 1 | … | … |
| Inner Regional | 3.38 (1.49, 7.65) | 1.41 | 0.004 |
| Outer regional and beyond | 5.47 (1.4, 21.34) | 3.8 | 0.014 |
|  |  |  |  |
| **Index of relative socioeconomic disadvantage quintile**^✝^ |  |  |  |
| 1-2 (Most disadvantaged) | 1 | … | … |
| 3-4 | 1 (0.47, 2.16) | 0.39 | 0.995 |
| 5-6 | 1.04 (0.32, 3.37) | 0.62 | 0.942 |
| 7-8 | 0.91 (0.27, 3.1) | 0.57 | 0.882 |
| 9-10 (Least disadvantaged) | 1.18 (0.46, 3.01) | 0.56 | 0.732 |
|  |  |  |  |
| **Marital Status**^✝^ |  |  |  |
| Married/De Facto | 1 | … | … |
| Divorced/Widowed/Separated | 0.49 (0.29, 0.84) | 0.13 | 0.009 |
| Never Married | 0.48 (0.22, 1.08) | 0.2 | 0.075 |
|  |  |  |  |
| **Num. Elixhauser comorbidities in year prior** | 1.18 (1.04, 1.34) | 0.08 | 0.011 |
|  |  |  |  |
| **Mental health Ambulatory use in year prior (per 10 days)** | 0.92 (0.87, 0.97) | 0.03 | 0.003 |
|  |  |  |  |
| **Involuntary mental health admissions in year prior (per 10 admissions)** | 0.94 (0.85, 1.06) | 0.05 | 0.312 |
|  |  |  |  |
| **Emergency department presentations in year prior  (per 10 presentations)^$^** | 1.25 (0.99, 1.6) | 0.15 | 0.065 |
|  |  |  |  |
| **History of depression** | 0.58 (0.31, 1.06) | 0.18 | 0.077 |
|  |  |  |  |
| **History of drug or alcohol abuse** | 0.91 (0.51, 1.61) | 0.27 | 0.738 |
|  |  |  |  |
| **History of psychotic disorder** | 1 (0.54, 1.83) | 0.31 | 0.988 |
|  |  |  |  |
| **History of anxiety disorder** | 1.07 (0.61, 1.89) | 0.31 | 0.804 |
|  |  |  |  |
| **History of delirium** | 1.05 (0.66, 1.68) | 0.25 | 0.838 |
|  |  |  |  |
| **History of behavioural problems** | 1.88 (1.09, 3.23) | 0.52 | 0.023 |
|  |  |  |  |
| **History of personality disorders** | 1.8 (0.9, 3.59) | 0.63 | 0.096 |
| ^✝^ “Unknown” category excluded from output | | | |
| ^$^ To allow for model convergence, if someone had over 100 presentations to ED in a single year, we treated them as having at most 101 presentations in that year. | | | |
